# Targeting *Leishmania donovani* Sphingosine Kinase 1 using PF-543 enhances immune response and limits parasite load

**DOI:** 10.1371/journal.pntd.0013102

**Published:** 2026-03-20

**Authors:** Evanka Madan, Ruby Bansal, Jhalak Singhal, Nishant Joshi, Monika Saini, Sadat Shafi, Aashima Gupta, Shailja Singh

**Affiliations:** 1 Special Centre for Molecular Medicine, Jawaharlal Nehru University, New Delhi, India; 2 Department of Biotechnology and Research, Sir Ganga Ram Hospital, New Delhi, India; 3 Amity institute of virology and immunology, Amity University, Noida, India; 4 Eli Lilly Services India Private Limited, Bengaluru, Karnataka, India; 5 Department of Pharmacology, School of Pharmaceutical Education and Research, Jamia Hamdard, New Delhi, India; Institute of Postgraduate Medical Education and Research, INDIA

## Abstract

**Background:**

Sphingosine-1-phosphate (S1P) is a bioactive lipid mediator regulating apoptosis, proliferation, and immune responses. While S1Ps presence in *Leishmania donovani* phagolysosomes has been reported, the role of sphingosine kinases, especially SphK1, in parasite survival and host immune modulation remains underexplored. This study investigates the molecular and functional role of *L. donovani* SphK1 (*Ld*SphK1) and evaluates the antileishmanial potential of PF-543, a specific SphK1 inhibitor.

**Methods:**

*Ld*SphK1 and human SphK1 (*rh*SphK1) were cloned, expressed in *E. coli*, purified, and analyzed by SDS-PAGE. Enzymatic activity and inhibition by PF-543 were assessed using NBD-S1P-based fluorometric assays. Protein-ligand interactions were analyzed using Microscale Thermophoresis (MST) and validated *in silico* docking studies, which identified key species-specific differences in the inhibitor’s active site. *Leishmania* promastigotes overexpressing *Ld*SphK1 were studied via confocal microscopy, and their viability and infectivity were assessed *in vitro*. THP-1 macrophages infected with *L. donovani* were treated with PF-543 alone or with Amphotericin B and analyzed by MTT assay, RT-PCR, Giemsa staining, ELISA and immunoblotting. *In vivo* efficacy was tested in *L. donovani*-infected Swiss mice.

**Results:**

*rLd*SphK1 (~102 kDa) and *rh*SphK1 (~50 kDa) were enzymatically active and significantly inhibited by PF-543. MST demonstrated specific, measurable binding of PF-543 to both orthologues (KD ~ 29μM under identical experimental conditions). In *L. donovani* SphK1 overexpressor *(Ld*SphKa) promastigotes, PF-543 inhibited SphK1 activity and reduced parasite infectivity, more than in wildtype *L. donovani* promastigotes. Notably, PF-543 treatment reduced parasite infectivity *in vitro*, lowered amastigote load by ~40%, and promoted a pro-inflammatory cytokine shift (↑IL-12, ↑ TNF-α, ↓ IL-10). Inhibition of ceramide synthesis and S1P supplementation revealed that S1P rescues ceramide-induced parasite death, implicating SphK1 in parasite survival. PF-543 and Amphotericin B demonstrated synergistic anti-parasitic effects both *in vitro* and *in vivo*, with >90% reduction in parasite burden in mice.

**Conclusion:**

PF-543 exerts moderate direct inhibition of parasite SphK1 while prominently modulating host SphK1-dependent immune and apoptotic pathways, collectively restricting *Leishmania* survival. Rather than functioning as a parasite-selective inhibitor, PF-543 acts as a dual host–parasite modulator. These findings provide proof-of-concept evidence that simultaneous targeting of sphingolipid signalling in both host and parasite can enhance anti-leishmanial efficacy and support further exploration of SphK-based combination therapeutic strategies.

## Author summary

Leishmaniasis, a neglected tropical disease, has limited available treatments and is becoming more resistant to medications. In this study, we explored the therapeutic potential of PF-543, a potent Sphingosine Kinase 1 (SphK1) inhibitor (demonstrated anticancer effects in various preclinical models) against *Leishmania donovani*. We successfully cloned and purified both *Leishmania* and human SphK1 proteins and confirmed PF-543 binding through biochemical and biophysical and supporting computational *(in silico)* analyses. Overexpression of *Ld*SphK1 in parasites enhanced their survival and infectivity. *In vitro,* PF-543 treatment of infected macrophages decreased amastigote burden, shifted cytokine profiles towards a pro-inflammatory state, and enhanced host cell apoptosis. Notably, PF-543 acted synergistically with Amphotericin B, the current clinical drug, both *in vitro* and *in vivo* in Swiss mice, drastically lowering parasite burden. This study identifies SphK1-dependent signalling as a biologically relevant axis at the host–parasite interface and highlight PF-543 as a proof-of-concept dual-acting modulator rather than a parasite-selective drug. This work supports host-directed and combination therapeutic strategies as promising avenues for improving leishmaniasis treatment.

## Introduction

Leishmaniasis is a neglected tropical disease caused by intracellular protozoa parasite *Leishmania* [1]. It invades macrophages and selectively impairs host’s critical signaling pathways for its successful intracellular growth and proliferation [2]. In particular, alteration in lipid metabolic pathways, lipid relocation, accumulation and mitochondrial dysfunction during *Leishmania* infection has been proven to be a critical step in the progression of disease [3].

Decrease in efficiency of current anti-leishmanial agents, increase in cost and complexity of curing *Leishmania*, increased parasitic resistance to the chemicals and limited number of available anti-leishmanial drugs has spur the research for novel approaches [4] Pentavalent antimonials (sodium stibogluconate and meglumine antimoniate), liposomal amphotericin B, Miltefosine and Paromomycin are the current drugs for Visceral Leishmaniasis [5,6]. However, resistance has been seen in endemic regions, where these drugs are extensively used, with risk of serious cardiotoxicity, toxic effects on the kidneys, leading to death [6,7]. The need for novel, effective, and safer therapeutic alternatives is therefore urgent.

In this context, targeting host or parasite-encoded enzymes has emerged as the promising strategy for identifying new anti-leishmanial agents. For the first time, we explored the potential of PF-543, a potent Sphingosine Kinase (SphK) inhibitor to modulate Sphingosine-1-phosphate (S1P) signalling, and disrupt *Leishmania* infection. Interestingly, evidences have suggested a key role for SphK in several lung pathologies, including lung fibrosis, pulmonary artery hypertension, asthma, ischemia reperfusion injury [8–12]. Recent studies have also implicated S1P signalling in the biology of *Leishmania donovani,* especially within phagolysosomes [13]*.*

Humans (host) express two sphingosine kinase isoforms, SphK1 and SphK2, with distinct regulatory functions [14,15]. SphK1 is more responsive to stimuli and increasingly regarded as a favourable therapeutic target compared to the more constitutively active SphK2 [16,17]. In contrast, *Leishmania donovani* (parasite) encodes a single sphingosine kinase homolog (commonly referred to as SphK or *Ld*SphK1), which shares partial sequence identity (~26% over ~58% of the sequence towards the C-terminus) and structural similarity (RMSD 0.585) with human SphK1 [18,19]. Our study focuses on modulating this parasite SphK1.

We investigate PF-543, a selective and potent inhibitor of SphK1 originally developed for anticancer applications [20], for its ability to modulate sphingosine-1-phosphate (S1P) signalling and disrupt *Leishmania* infection. Both host and parasite harbour SphK1, making PF-543 a unique dual-target inhibitor. Importantly, inhibition of host SphK1 appears safe due to compensatory activity of SphK2 [21,22], minimizing side effects and reducing resistance risks by targeting a host rather than parasite protein.

In this study, we cloned and expressed both *Leishmania* and human SphK1, validated PF-543 binding through Microscale Thermophoresis (MST), comprehensively validated *in silico* docking, and enzyme kinetics, and assessed the functional impact of modulating SphK1 in parasite survival, infectivity, inflammatory responses, and combined efficacy with amphotericin B both *in vitro* and *in vivo.*

Usage of Amphotericin B liposome as anti-leishmanial is already published [23] and patented [24]. PF-543, the most potent SphK1 inhibitor developed by Pfizer, is not yet known to be an anti-leishmanial drug. Here, for the first time, we report the anti-leishmanial potential of PF-543, both as standalone agent and in combination with Amphotericin B as a new anti-leishmanial formulation.

These findings not only provide insight into host-parasite interactions but also discusses that PF-543 targets both host and parasite SphK1 isoforms, but with differential binding affinities and expression contexts. Detailed comparison between the sequence, structure, and binding selectivity of host vs. parasite SphK1, corroborated by newly validated docking and kinetic data that reveals crucial species-specific differences in the inhibitor’s binding pocket. The integration of *in silico*, biochemical, and *in vivo* findings further underscore the translational potential of PF-543 as a prototype for next-generation SphK1-directed therapeutics against *Leishmania*.

## Methods

### Ethics statement

Animal studies were performed following CPCSEA guidelines and approved by the Institutional Animal Ethics Committee (IEAC, (JNU/IAEC/2020/19)) of Jawaharlal Nehru University. Swiss mice were obtained from the Central Laboratory Animal Resources, JNU, New Delhi, and maintained under standard conditions.

### In silico search for SphK1 like protein in *Leishmania donovani*

An *in-silico* search was conducted in the Kinetoplastid database TriTrypDB (www.tritrypdb.org) to identify any homologous protein similar to prokaryotic SphK1 in *Leishmania donovani* [25]. Sequence alignments and identity computations were conducted using ClustalW [26]. Homologues of SphK1 from *L. donovani*, along with other prokaryotes and eukaryotes were analyzed using the Molecular Evolutionary Genetics Analysis (MEGAX) tool [27]. The evolutionary trajectory was determined using the Neighbor-Joining method. The bootstrap consensus tree was generated from replicates and the evolutionary distances were calculated using the Poisson correction method. Pymol was utilized to align the two structures and compute the RMSD post-alignment [28].

### In-silico docking studies

The protein structure of hSphK1 (Uniprot ID- Q9NYA1), a protein derived from *H. sapiens*, was retrieved from the protein data bank [29,30]. The *Leishmania major* Sphingosine Kinase A sequence, as previously characterized by Zhang et al. [31], was used as a query to identify the corresponding orthologue in the *L. donovani* genome via a BLASTp search of the TriTrypDB database [32]. Phyre2 web server was used for homology 3D protein modelling of the *Ld*SphK1 protein and Procheck sever was used for Ramachandran plot analysis of the model [33,34]. The stereochemical quality of the resulting model was then comprehensively validated using the MolProbity server [35,36]. The Swiss PDB viewer and ChemBio Draw ultra 3D software were used to optimize the structure of proteins and ligands [37,38]. The process of molecular docking between molecule PF-543 and hSphK1 was conducted using Autodock version 4.2 and Cygwin terminal version 3.1 software [39,40]. The residues located in the vicinity of the binding pocket were selected to create a grid (with a spacing of 0.375) centred at coordinates X-51.488, Y-53.406, Z- -1.49 for hSphK1 and X-4.021, Y- -21.535, Z- 15.254 for *Ld*SphK1 respectively. This grid was used to facilitate the binding of the ligand. The docking data were further analyzed and visualized using the PLIP, Ligplot+ version 2.2, Discovery Studio version 19.1.0, and Pymol version 2.3.2 software tools [28,41–43].

Homologs of SphK1 from *L. donovani* and other representative kinetoplastids and mammals were retrieved from TriTrypDB and NCBI, respectively. The amino acid sequences were aligned using ClustalW. The phylogenetic tree was reconstructed using the Maximum Likelihood (ML) method in MEGA XII [44]. The best-fit substitution model was first determined by MEGA’s model selection tool, which identified the Jones-Taylor-Thornton (JTT) model with a Gamma distribution (+G), a proportion of invariable sites (+I), and observed amino acid frequencies (+F) as the optimal model based on the Bayesian Information Criterion (BIC). The tree was inferred using the Subtree-Pruning-Regrafting (SPR) heuristic method. The reliability of the tree topology was assessed by bootstrap analysis with 1000 replicates.

### Cloning and purification of recombinant LdSphK1

Nucleotide sequence encoding full length sequence of *L. donovani* SphK1 (1–2808 bp) was selected for recombinant protein generation as a fusion protein with an N-terminal 6x- Histidine-tag using the vector pET-28 a(+) vector. DNA fragment for the gene cloning was PCR amplified from genomic DNA of *L. donovani* using the following primer pair: *Ld*Sphk1_BamHI_FP 5’-GATGGGATCCATGCACTCTTTCACCTCCAA-3’ and *Ld*Sphk1_XhoI_RP 5’-ATTCTCGAGCTACCCACTGCGCACGAGCT-3’ with Phusion High-Fidelity DNA Polymerase (Thermo Scientific, US). The amplified DNA fragment was purified with QIAquick Gel Extraction Kit (Qiagen) using the manufacturer’s protocol. Purified *Ld*Sphk1 insert and the expression vector pET-28 a(+) were digested with BamHI/XhoI restriction enzymes (New England Biolabs, UK), and were ligated overnight at 16°C using T4 DNA ligase (New England Biolabs, UK). The ligation mix was transformed into *E. coli* DH5-α competent cells (*E.Coli* DH5-α cells were obtained from ThermoFisher Scientific, USA Catalog number C0112) and positive clones were screened by colony PCR followed by confirmation of the cloned plasmid with BamHI/XhoI restriction digestion and further transformed in Rossetta *E. coli* (Rossetta *E. coli* were obtained from Sigma-Aldrich, USA with Catalog number 71397) expression strain for *Ld*Sphk1 recombinant protein expression. For recombinant protein induction, the cells were induced with 1mM IPTG for 4 hours at 37°C in Terrific broth. Purification of recombinant protein was achieved by affinity chromatography.

The *rLd*SphK1 protein was eluted after addition of 150mM imidazole solution. The eluted fractions were analysed by running on 12% SDS PAGE and the fractions containing pure protein were pooled and concentrated using a centricon device, followed by buffer exchange with PBS. Since most of the protein was localized in the inclusion bodies, we purified the protein from inclusion bodies under denaturation conditions. Briefly, inclusion bodies were obtained after sonication and subsequent centrifugation at 13,000 rpm and solubilized in urea buffer composed of 8M urea, 20mM Tris, 250mM NaCl with pH 8.0 and subsequently incubated with Ni-NTA beads (Qiagen, Germany) overnight for binding. The beads were then loaded in gravity flow column (Qiagen, Germany). Further, *Ld*SphK1 protein bound with the beads was then eluted by 10mM, 25mM, 50mM, 100mM, 250mM and 500mM imidazole solutions prepared in urea buffer. Eluted fractions containing pure protein were pooled and refolded by dialyzing against refolding buffer composed of 100mM Tris, 20% Glycerol, 250mM L-Arg, 1mM EDTA, 1mM GSG and 0.5mM GSSG with pH 8.0. Refolded protein was reconstituted in PBS (pH 7.4) and concentrated using centricon tubes with 10kDa cut-off (Merck, Germany).

### Purification of recombinant humanSphK1

CDS encoding full-length SphK-1 (1155 bp) (Gene ID-8877) was amplified using the following primers:

NheI_FP: 5′-AATGCTAGCATGGATCCAGCGGGCGGCCCC-3′ and

XhoI_RP: 5′-TGGCTCGAGCTATAAGGGCTCTTCTGGCGGTG-3′.

The amplified DNA fragment was cloned between NheI and XhoI restriction sites of the pET-28a (+) expression vector, and the recombinant plasmid was transformed into *E. coli* strain BL21 (ʎDE3) gold [45]. The *rhSphK1* protein was eluted after addition of 100mM, 250mM and 500mM imidazole solution. Overexpression of 6 × His-SphK1 (*rSphK1*) was induced with 1mM IPTG (Sigma-Aldrich) at an optical density (OD600) of 0.6, for 4h at 37°C. The protein was purified using Ni-NTA agarose resin (Qiagen). Briefly, Rosetta (DE3) competent cells, derivatives of *E. coli* BL21 obtained from Sigma-Aldrich (Catalog No. 71397), were used to enhance *rh*SphK1 expression by compensating for rare codon usage in *E. coli*. Bacterial cells were harvested by centrifugation at 6000 × g for 15 minutes and lysed by sonication in lysis buffer containing 50 mM Na₂HPO₄ (pH 7.4), 200 mM NaCl, 500 μg/mL lysozyme (Sigma-Aldrich), and 1mM Phenylmethylsulfonyl Fluoride (PMSF; ThermoFisher Scientific). The bacterial lysate was centrifuged at 10,000 × g for 20 min, and the supernatant obtained was loaded onto a Nickel-Nitrilotriacetic Acid (Ni-NTA) agarose resin (Qiagen)-packed column along with 10 mM imidazole, for 4h at 4°C with mild agitation. The protein was eluted with a continuous imidazole gradient of 25mM, 50mM, 100mM, 250mM, and 500mM. The protein purification was validated by 12% SDS-PAGE, followed by immunoblotting with anti-His tag antibody (raised in Swiss mice (obtained from the Central Laboratory Animal Resources, Jawaharlal Nehru University, New Delhi) using standard protocol approved by IAEC, JNU).

### Microscale thermophoresis (MST)

To validate the biophysical interaction between *rLd*SphK1 or *rHuman* SphK1 with PF-543 and analyze the binding and kinetics, Microscale Thermophoresis (MST) was performed in NanoTemper Monolith NT.115 instrument. Briefly, 20μM of *rLd*SphK1 or *rh*SphK1 in HEPES-NaCl buffer, pH 7.4 was labelled with 30μM Lysine reactive dye (5ul diluted in 200ul *rLd*SphK1 or *rh*SphK1 protein) using NanoTemper’s Protein Labelling Kit RED-NHS (L001, NanoTemper technologies, Germany) and incubated in the dark for 30 minutes at RT. Following incubation, the labelled *rLd*SphK1 or *rh*SphK1 protein along with the buffer was passed through an equilibrated column (provided in the kit). Fractions of the labelled protein were eluted followed by fluorescence count. Fluorescence counts ranging between 250–450 were taken. 5 µM PF-543, diluted in HEPES/0.05% Tween-20, was titrated against the constant concentration of the labelled *rLd*SphK1 or *rh*SphK1 protein. Pre-mixed samples were incubated for 15 minutes before centrifugation at 8000 rpm for 10 minutes. The samples were filled into the capillaries treated with standards (K002 Monolith NT.115) and thermophoretic mobility was determined. All experiments were performed at RT, at 40% MST power and 20% LED power. Data evaluation was performed with the Monolith software (Nano Temper, Munich, German). Dissociation constants (K_D_) were derived from concentration-dependent thermophoretic signal changes. It is emphasized that MST-derived K_D_ values are context-dependent and reflective of experimental conditions**,** and do not replace previously reported enzymatic IC₅₀ values for mammalian SphK1 obtained using optimized biochemical assays [45].

### NBD-Sph assay

A fluorescent sphingosine analog, omega (7-nitro-2–1, 3-benzoxadiazol-4-yl [2S,3R,4E]-2-amino octadec-4-ene-1,3-diol [NBD–Sphingosine (NBD-Sph); Avanti Polar Lipids]) was used as a substrate. Conversion of NBD-Sph to NBD-S1P was evaluated, where NBD-SIP served as the measurable end product of sphingolipid metabolism. Towards this, *rLd*SphK1 protein was incubated with 10μM NBD-Sph, at 37°C for 60 min. Following incubation, the reaction was terminated by addition of RIPA lysis buffer and lipids were extracted using a modified chloroform–methanol extraction protocol. Briefly, the reaction mixture was thoroughly mixed with 260μL methanol and 400μL chloroform:methanol (1:1) followed by addition of 16μL of 7 M NH4OH, 400μL chloroform, and 300μL of 1.5 M KCl. The mixture was centrifuged at 17,000 × g for 5 min to allow phase separation.

A 100μL aliquot of the upper aqueous phase containing NBD-S1P was transferred to a black 96-well flat-bottom plate (Corning). Fluorescence intensity was measured at excitation/emission wavelength of 485 nm/530 nm using Varioskan LUX Multimode Microplate Reader (ThermoFisher Scientific). The fluorescence intensity directly correlated with NBD-S1P formation.

### Biochemical characterization of purified rLdSphK and rHumanSphK

The inhibitory effect of PF-543 on the *rLd*SphK1 catalytic activity was evaluated in a dose-dependent manner (0.01–5µM). The recombinant protein (200 ng) was pre-incubated with different concentrations of PF-543 (0.01μM to 5μM) for 45 min., followed by initiating the reaction in a buffer containing 0.05% Triton X-100, 1mM ATP (saturating concentration), 2% DMSO and 10μM NBD Sphingosine. The fluorescence intensity of NBD-S1P was measured as described above. The results represented a maximum inhibition at 5μM of PF-543, while the protein concentration was kept constant at 200ng.

For kinetic analysis, varying concentrations of NBD-Sphingosine (2–20µM) were incubated with 200ng *rLd*SphK1 in the presence of 200µM ATP, a concentration approximating reported Km values for ATP in SphK1, to allow accurate determination of kinetic parameters. Readings were taken real-time after every 5 sec for 15–20 min at an excitation/emission wavelength of 490/530 nm. Standard curve of SIP was plotted. The enzymatic activities were calculated by plotting fluorescence/time vs substrate concentration followed by plotting of fluorescence/pmoles (velocity) vs substrate concentration. Finally 1/Vmax vs 1/[S] was plotted to calculate Km and Vmax according to Lineweaver-Burk plots at 37°C (Lineweaver and Burk, Science Direct, 2011).

### *Leishmania* cell culture

*Leishmania donovani Bob* promastigotes (*Ld*Bob strain/MHOM/SD/62/1SCL2D, originally obtained from Dr Stephen Beverley (Washington University, St. Louis, MO) were cultured at 26°C in M199 medium (Sigma-Aldrich, USA), supplemented with 100 units/ml penicillin (Sigma-Aldrich, USA), 100µg/ml streptomycin (Sigma-Aldrich, USA) and 10% heat-inactivated fetal bovine serum (FBS; Biowest). Whole cell lysate of promastigote was prepared by freeze–thaw cycles as described previously [46].

For axenic amastigotes, the axenically cultured forms grew optimally at a temperature of 32–33°C in a growth media with pH of 5.4. The axenic amastigotes were prepared according to the standard protocol [47–49] Briefly, the late-log promastigotes were adapted in an acidic media (RPMI-1640/25 mM 2-(N-morpholino) ethane sulfonic acid (MES)/pH 5.5), at 26°C. These parasites were then grown in RPMI-1640/MES/pH 5.5 at 37°C with 5% CO2, mimicking the acidic and increased temperature conditions of the macrophage phagolysosomal compartment. Axenic amastigotes were microscopically examined to confirm their conversion from promastigotes to amastigotes as per the previous report [47–49].

### Transient transfection of the parasite *Leishmania*

*Leishmania donovani* Bob strain (*Ld*Bob strain/MHOM/SD/62/1SCL2D) promastigotes were transfected with pXG-GFP-SphKa OE plasmid which harboured the neomycin resistance cassette using electroporation technique as described previously [50] with a Bio-Rad Gene Pulser device and cultured at 26°C in M199 medium (Gibco, Thermofisher scientific USA), supplemented with 100 units/ml penicillin (Sigma-Aldrich, USA), 100µg/ml streptomycin (Sigma-Aldrich, USA) and 10% heat-inactivated fetal bovine serum (FBS; Gibco, USA). A final concentration of 250μg/mL of G418 (Sigma-Aldrich, USA) was used to select transfected parasites.

### THP-1 cell culture and infection

THP-1 cells, an acute monocytic leukaemia-derived human cell line (American Type Culture Collection, Rockville, MD), were maintained in RPMI-1640 (Sigma-Aldrich, USA) medium supplemented with 10% heat-inactivated FBS (Biowest, UK), 100 units/ml penicillin and 100 μg/ml streptomycin at 37°C and 5% CO_2_. Cells (10^6^ cells/well) were treated with 50 ng/ml phorbol-12-myristate-13-acetate (PMA) (Sigma-Aldrich, USA) for 48h to induce differentiation into macrophage-like-cells before infection. Cells were washed once with phosphate-buffered saline (PBS) and incubated in RPMI medium (Sigma-Aldrich, USA), 10% heat-inactivated FBS, 100 units/ml penicillin and 100μg/ml streptomycin, before infection. To carry out *in vitro* infection assays, late stationary phase promastigotes (WT) were used at a ratio of 20 parasites per macrophage. *Leishmania*-infected macrophages were incubated at 37°C in a 5% CO_2_-air atmosphere for 4h to allow the establishment of infection and proliferation of intra-macrophage parasites. The cells were then washed five times with PBS to remove non-adherent extracellular parasites. After that, the cells were incubated in RPMI medium at 37°C in a 5% CO_2_-air atmosphere for 48h.

### Infectivity assay

THP-1 cells (1 × 10^6^ cells/well), treated with 50 ng/ml of PMA (Sigma-Aldrich, USA**)** were seeded on glass coverslips in 6-well plates for 48h. They were infected and simultaneously treated with inhibitors as described above, and the intracellular parasite load (mean number of amastigotes per macrophage) was visualized by Giemsa staining as described previously by Pawar *et al* [51] and expressed as a percentage of the blank controls without drug. At least 10 fields were counted manually for each condition to determine the average number of parasites per macrophage.

### qRT-PCR for gene expression analysis

Total RNA from infected macrophages was isolated using the TRIZOL reagent (Sigma-Aldrich, USA) and its concentration was determined by Nanodrop (Thermo Fischer, USA). cDNA was prepared from one microgram of RNase-free DNase treated total RNA using first-strand cDNA Synthesis Kit (Thermo Fischer Scientific, USA), as per manufacturer’s instructions, using random hexamer primers. The resulting cDNA was analyzed by quantitative real-time (qRT-PCR) RT-PCR (Applied Biosystems, 7500 Fast Real-Time PCR System, CA, USA) with gene-specific primers using PowerUp SYBR Green PCR Master Mix (Thermo Fisher Scientific, USA). The details of the primers (sequences and annealing temperatures) are given in **S1 Table**. Thermal profile for the real-time PCR was amplification at 50°C for 2 min followed by 40 cycles at 95°C for 15 sec, 60°C for 1 min and 72°C for 20 sec. Melting curves were generated along with the mean C_T_ values and confirmed the generation of a specific PCR product. Amplification of RNU6AP (RNA, U6 small nuclear 1; THP-1 cells) was used as internal control for normalization. The results were expressed as fold change of control (Uninfected samples (RNU6AP)) using the 2^-ΔΔ*CT*^method. All samples were run in triplicates, including a no-template (negative) control for all primers used.

### Western blotting

Western blot analysis was done as described previously by Darlyuk et al., 2009 [52]. Briefly, protein was isolated from *Leishmania* infected macrophages by resuspending cell lysates in RIPA buffer. Before lysis, adherent cells were placed on ice and washed with PBS. Macrophages were scraped in the presence of RIPA lysis buffer containing 1% NP-40, 50 mM Tris-HCl (pH 7.5), 150 mM NaCl, 1 mM EDTA (pH 8), 10 mM 1,10-phenanthroline and phosphatase and protease inhibitors (Roche). After incubation, lysates were centrifuged for 15 min to remove insoluble matter.The proteins in the lysates were quantified, 80μg of the lysate was boiled (95˚C) for 5 min in SDS sample buffer and was subjected to electrophoresis on a 10% SDS-polyacrylamide gel. Proteins were then transferred onto nitrocellulose (NC) membrane using an electrophoretic transfer cell (Bio-Rad Laboratories, USA) at RT. The membrane was washed with 1 × TBST solution three times and blocked with 5% BSA for 2h at RT. The blocked membrane was washed again in TBST solution three times and incubated overnight at 4 °C with primary monoclonal antibody against Caspase-9 (Cell Signaling Technology, #9508) diluted in TBST containing 5% BSA. The blots were subsequently incubated with HRP-conjugated secondary antibody (1:3000 dilution) prepared in 5% BSA in TBST for 2h at RT. Enhanced chemiluminescence reaction was used for the detection of the blot. The results were expressed as fold change and quantitated by using AlphaEaseFC image analysis software (Alpha Innotech). The data were expressed as mean ± SD of three independent experiments, and the representative image of one experiment is shown.

### Localization of LdSphK1 in *L. donovani* promastigotes

The amino acid sequence of *Ld*SphK1 was analyzed for the presence of transmembrane domains using the DeepTMHMM server. Further, for detection of SphK1 localization in parasites, 5*10^6^ log phase *Leishmania donovani* promastigotes were plated on poly-L-Lysine coated glass coverslips followed by treatment with 500nM PF-543 diluted in incomplete M199 medium for 6h at 22°C in dark. After 6h of treatment, the cells were washed with 1XPBS, fixed (PBS, 4% formaldehyde, 30 min) and permeabilized (PBS, 0.5% Triton X-100, 5 min). Slides were blocked (1% BSA, 1XPBS, overnight at 4°C or for 30 minutes on rocker, RT). Next day blocked cells were incubated with mice raised anti-SphK1 primary antibody (1:500) for 2 hours at RT. The cells were then washed three times with blocking buffer followed by incubation with Alexa 488 conjugated secondary antibody for 1 hour at RT. The coverslips were mounted on glass slides with antifade DAPI solution for visualization under a confocal laser scanning microscope (Olympus FluoView FV1000 with objective lenses PLAPON ×60 O, NA-1.42) at an excitation wavelength of 556 nm. Images were processed via NIS-Elements software version 4.50. The mean fluorescence intensities were plotted using GraphPad Prism version 8.0. The experiment was performed thrice.

### Checkerboard assay

Checkerboard assays were put to evaluate the effects of the combination of Amphotericin B (anti-fungal agent) and PF-543 (Sphingosine Kinase inhibitor) against the *Leishmania* parasite. For this, log-stage promastigotes (42–44h) maintained in M199 media were dispensed in a 96-well plate. Amphotericin B was added vertically at different concentration ranges while PF-543 was added horizontally at different drug concentration ranges in 8*8 format. As a result, the checkerboard consists of columns and rows in which each of the well along the x-axis contains drug Amphotericin B at different concentrations (12.5nM, 30nM, 50nM and 100nM) and that along the y-axis contains PF-543 at different concentrations (150nM, 250nM, 500nM and 2µM). The plate was incubated at 25°C in a humidified chamber for 48h. The fractional inhibitory concentration (FIC index = FIC A + FIC B, where FIC A is the IC_50_ of drug (A) in combination/IC_50_ of drug A alone, and FIC B is the IC_50_ of drug B in combination/IC_50_ of drug B alone) of each drug was calculated and plotted as an isobologram. A straight diagonal line with an FIC index equal to 1 indicates an additive effect between drug A and drug B, a concave graph below the diagonal with an FIC index of less than 1 indicates a synergistic effect, and a convex curve above the diagonal with an FIC index of more than 0 indicates antagonism [53–55].

### Estimation of reduced (GSH) and oxidized (GSSG) glutathione levels

The estimation of glutathione levels in reduced and oxidized forms was performed in accordance with the previously documented protocol [56]. Briefly, *Leishmania* infected macrophages were incubated with varying concentrations of SphK inhibitors for 24h and lysate was prepared. For GSH estimation, the lysate was added to an assay mixture containing 0.1 mM sodium phosphate buffer (pH 8) and o-phthalaldehyde (1 mg/ml in methanol, Sigma Aldrich, USA) and incubated at RT for 15 min. GSH level was estimated by monitoring fluorescence change at 365Exc/430Emi using a microplate reader. GSSG was also estimated using the same method, however, the lysate was incubated first with N-ethylmaleimide (Sigma Aldrich, USA) in the dark for 5 min, to block the GSH to GSSG oxidation. Results plotted as redox index (GSH/GSSG) ratio are the mean of duplicates.

### Animal studies

Swiss mice were housed under standard conditions of food, temperature (25 ± 3 °C), relative humidity (55 ± 10%) and illumination (12h light/dark cycles) obtained from the Central Laboratory Animal Resources, Jawaharlal Nehru University, Delhi.

For experimental assays, mice were divided into the following groups (n = 3 per group): Uninfected control group, Infected untreated control group, Infected + PF-543 (10 mg/kg), Infected + Amphotericin B (2 mg/kg), Infected + Combination therapy (PF-543 2 mg/kg + Amphotericin B 0.4 mg/kg). Mice in treatment groups were infected with *L. donovani* 24h prior to drug administration. Here-in, 10mg/Kg of PF-543 and 2mg/Kg of Amphotericin B were used as per the reported literature (Neil MacRitchie et al. Cell Signal; 2016 [57], EM Moore and D N Lockwood. J Glob Infect Dis; 2010, [6]). All drugs were administered intraperitoneally (i.p.) once daily for three consecutive days. PF-543 monotherapy: 10 mg/kg/day, Amphotericin B monotherapy: 2 mg/kg/day, Combination therapy: 2 mg/kg/day PF-543 + 0.4 mg/kg/day Amphotericin B. Drug doses were prepared freshly in 1 × PBS and administered in a final volume of 100 µL per 30 g mouse. Briefly, to prepare 10mg/Kg/day of PF-543, 0.3mg of PF-543 powder was dissolved in 1ml of 1XPBS (5.9mM). 100µl of prepared PF-543 in 1XPBS suspension was then given to each mouse weighing 30gm. To prepare 2mg/Kg/day of Amphotericin B, 0.06mg of Amphotericin B powder was dissolved in 1ml of 1XPBS (650µM). Further 100µl of this prepared Amphotericin B in 1XPBS suspension was then given to each mouse weighing 30gm. For constituting the synergistic effect of two drug partners; 2mg/Kg (1mM) of PF-543 and 0.4mg/Kg (0.12µM) of Amphotericin B were prepared in 1XPBS and given to a mouse weighing 30gm. To prepare 2mg/Kg/day of PF-543; 0.06mg of PF-543 was dissolved in 1ml of 1XPBS (1mM). Further, 100µl of this prepared PF-543 suspension in 1XPBS was given to each mouse weighing 30gm. To prepare 0.4mg/Kg/day of Amphotericin B; 0.012mg of Amphotericin B powder was dissolved in 1ml of 1XPBS (0.12µM). Further, 100µl of this prepared Amphotericin B suspension was given to each mouse weighing 30gm. All treated mice were monitored for adverse effects (body weight, behaviour, and survival) throughout the treatment period. No significant toxicity was observed at the administered doses, consistent with published reports of the tolerability of both compounds in mouse models. Further, evaluation of the immunomodulatory potential was done using cytokine profiling in *Leishmania* infected Swiss mice treated with PF-543 and Amphotericin B alone and simultaneously followed by determination of parasite infectivity using *in-vitro* parameters. To evaluate the parasitaemia and expression of inflammatory markers in infected mice upon drug administration, spleen was harvested from each group of mice and qRT-PCR analysis was performed using primers specific for inflammatory markers; TNF-α and IL-10 and parasite specific kinetoplast minicircle gene; JW in PF-543 and Amphotericin B treated infected mice in respect to the untreated Swiss mice.

### Statistical analysis

Fold-expression (qRT-PCR and densitometric analysis), NBD-Sph and intracellular parasite burden were represented as mean ±SD. Each experiment was repeated three times in separate sets. Statistical differences were determined using Student’s unpaired 2-tailed *t*-test. All statistics were performed using GraphPad Prism Version 5.0 (GraphPad Software, USA). p ≤ 0.05 was considered significant [* (P < 0.01 to 0.05), ** (P < 0.001), *** (P < 0.0001), ns (P ≥ 0.05)].

## Results

### 1. Inhibition of SphK1 by PF-543 selectively impairs *Leishmania donovani* growth and reduces intracellular parasite load

**Fig 1:**
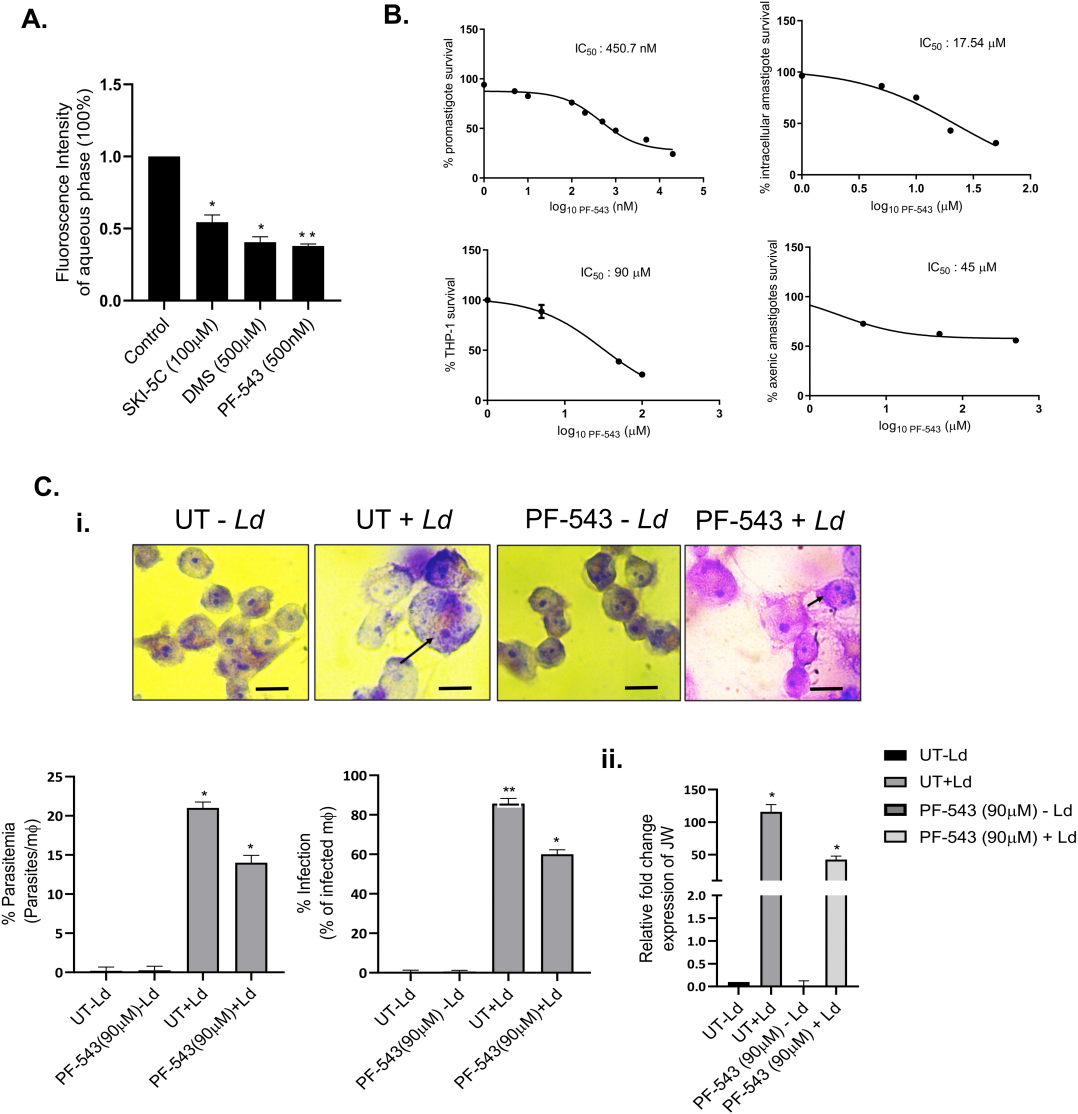
Inhibition of SphK1 by PF-543 selectively impairs *Leishmania donovani* growth and reduces intracellular parasite load. (A) Estimation of *Ld*SphK-1-mediated generation of NBD-SIP levels against SphK inhibitors in *L. donovani* promastigotes. *L. donovani* Bob promastigotes were cultured in 25 cm^2^ flasks followed by treatment with SphK inhibitors; PF-543, DMS and SKI-5C for 48h. Inhibition of SphK1 using these SphK inhibitors cause decreased S1P levels. SphK inhibitor treated promastigotes were resuspended in buffer containing fatty acid-free BSA (0.1% (w/v)) followed by resuspension in buffer containing BSA (1% (w/v)) and incubated with NBD-sphingosine (10μM) for 45 min at 37°C. Promastigotes incorporate NBD-sphingosine (NBD-Sph), which are phosphorylated by Sphingosine Kinase (SphK1) to NBD-S1P. Bar graph depicts ELISA-based S1P quantification in promastigotes treated with inhibitors. (B) Effect of SphK1 inhibitor; PF-543 in *Leishmania spp* and host macrophages. To evaluate the IC_50_ for PF543, approximately 6 × 10^3^ THP-1 and 5 × 10^4^ *Ld* Bob cells were seeded in each well of 96-well flat bottom plates and supplemented with RPMI and M199 media containing varying concentration of PF-543 (200µl/well) in each well. For intracellular amastigotes, 1 × 10^6^ THP-1 cells, treated with 50 ng/ml of phorbol 12-myristate 13-acetate (PMA) were seeded on glass coverslip in a 6-well plate for 48h. They were infected with late log-phase *L. donovani* promastigotes at 20:1 MOI and simultaneously treated with PF-543. The cells were further incubated for 2 days at 37°C and 5% CO_2_. To determine, the intracellular parasite burden (mean number of amastigotes per macrophage) were microscopically assessed using Giemsa staining. For axenic amastigotes, the axenically cultured forms grew optimally at a temperature of 32-33°C in a growth media with pH of 5.4. The IC_50_ were found to be 450.7nM, 17.54μM, 45μM and 90μM for *Leishmania donovani* promastigotes, intracellular amastigotes axenic amastigotes and THP-1 macrophages respectively. Each experiment was done in triplicates and repeated thrice. (C) Effect of SphK1 inhibition on parasite infectivity in THP-1 macrophages. THP-1 cells grown in RPMI medium were treated with SphK1 inhibitor; PF-543 for 24h followed by infection with *L. donovani* promastigotes at an MOI of 20:1 for 48h. (i) After 48h of infection, Giemsa staining was performed to assess the parasite burden. THP-1 cells were fixed, Giemsa stained and amastigotes were counted visually under 100X using light microscope (Scale bar - 20 µm). Virulence capacity was determined by calculating infectivity (upper panel) and parasitemia (lower panel). Untreated infected THP-1 cells were used as control. (ii) Total RNA was enriched using TRIzol. Infectivity was validated using qRT-PCR with primer specific marker JW as a molecular indicator. Expression of JW mRNA in infected cells inferred parasite load and represented as percentage infectivity. Data analysis was performed using the 2^-ΔΔ*CT*^method. The results are representative of three independent experiments. Statistical significance was quantified using the unpaired t-test. The results signify mean ± S.D with n = 3, *P < 0.05.

The activity of *Leishmania*-specific sphingosine kinase-1 (*Ld*SphK1) was assessed in promastigotes following exposure to three known Sphingosine Kinase inhibitors: PF-543 (500 nM), DMS (500µM), and SKI-5C (100µM) for 48 h (IC₅₀ values represented in **Fig 1A and** **S1****–****S2 Figs****).** To investigate the impact of SphK inhibitors; such as PF-543, DMS and SKI-5C on catalytic activity of SphK1 expressed by *Leishmania* promastigotes (*Ld*SphK1), we performed fluorometric assay based estimation of enzymatic activity using 7-nitro-2–1,3-benzoxadiazol-4-yl (NBD)-tagged sphingosine (NBD-sphingosine). The results demonstrated significant ~60% reduction of NBD-S1P levels in PF-543-treated *Leishmania* promastigotes compared to the untreated control **(Fig 1A)**. This observation validated the presence of an enzymatically active form of SphK1 in *Leishmania donovani* and confirmed that PF-543 effectively targets its catalytic activity.

To evaluate the anti-parasitic efficacy and host cytotoxicity profile of PF-543, dose–response analyses were carried out in different biological systems: *L. donovani* promastigotes (*Ld*Bob strain/MHOM/SD/62/1SCL2D), axenic amastigotes, intracellular amastigotes, and host THP-1 macrophages (an acute monocytic leukaemia-derived human cell line, American Type Culture Collection, Rockville, MD). Cytotoxicity was quantified using the MTT assay after 48h of treatment **(Fig 1B)**. It was found to be most effective against the promastigote stage of *Leishmania donovani* (IC₅₀ = 450.7nM), moderately effective against intracellular amastigotes (IC₅₀ = 17.54μM), and axenic amastigotes (IC₅₀ = 45μM), while demonstrating the least potency and cytotoxicity against host THP-1 macrophages (IC₅₀ = 90μM) (**Fig 1B**). These findings suggest that PF-543 possesses potent, stage-specific anti-leishmanial activity, with marked selectivity toward the parasite over host cells. PF-543 demonstrated moderate inhibition of promastigote SphK1 activity and exhibited micromolar-range IC₅₀ values against infective parasite stages, including intracellular amastigotes (>10µM), with modest selectivity (~2-fold) relative to host cells **(Fig 1B)**. Importantly, cytotoxicity towards host THP-1 macrophages remained higher (~90µM) than the intracellular amastigote IC₅₀ (~17µM), supporting a therapeutic window favouring host-mediated parasite control rather than direct parasiticidal activity reported. These findings indicate dual targeting potency towards *Leishmania* and host.

The pronounced susceptibility of extracellular promastigotes compared to intracellular amastigotes aligns with earlier studies demonstrating similar stage-dependent sensitivity differences across *Leishmania* species [58–60]. The reduced efficacy in the intracellular stage likely reflects the protective environment provided by the parasitophorous vacuole within host macrophages, which can limit drug access and potency.

To further discern whether the observed anti-parasitic effect was specific to SphK1 inhibition, the role of SphK2 was examined using ABC294640, a selective SphK2 inhibitor. Interestingly, ABC294640 exhibited comparatively higher cytotoxicity towards host THP-1 macrophages (IC₅₀ = 60μM) and intracellular amastigotes (IC₅₀ = 69.7μM) **(****S2 Fig**), underscoring that PF-543 selectively targets the SphK1 pathway with a more favourable therapeutic window.

Collectively, these findings establish PF-543 as a potent and selective inhibitor of *Leishmania donovani* SphK1, effectively impairing parasite growth while exhibiting limited host cell toxicity. This highlights SphK1 as a promising molecular target for anti-leishmanial drug development.

To further evaluate the effect of PF-543 on the intracellular survival of *L. donovani,* THP-1 cells were pre-treated with PF-543 (90µM) for 24h prior to infection with *L. donovani* at an MOI of 20:1. The intracellular parasite burden was assessed 48h post-infection using Giemsa staining and RT-PCR. Untreated- uninfected macrophages (UT-*Ld*) served as experimental controls whereas untreated, infected macrophages (UT + *Ld*) represented the baseline infection group. The UT + *Ld* group exhibited an infection rate of approximately 80%, confirming efficient infection under experimental conditions. In contrast, PF-543-treated infected macrophages (PF-543 + *Ld*) showed a marked reduction in intracellular infection, with only ~60% of macrophages harbouring amastigotes **(Fig 1Ci).** Upon comparing the clearance of parasitic load in PF-543 treated infected THP-1 cells (PF543 + *Ld*) with untreated infected THP-1 (UN + *Ld*), ~ 40% reduction in the number of parasites (amastigotes/macrophage) was detected per infected macrophage **(Fig 1Cii).**

Collectively, these findings support a dual-acting mode of action**,** wherein PF-543 induces moderate inhibition of parasite SphK1 while simultaneously modulating host cellular pathways that restrict intracellular parasite survival.

### 2. Expression and Purification of the recombinant *Leishmania* SphK1 (*rLdSphK1*) protein and its Functional Characterization in presence and absence of SphK1 inhibitor- PF-543

**Fig 2:**
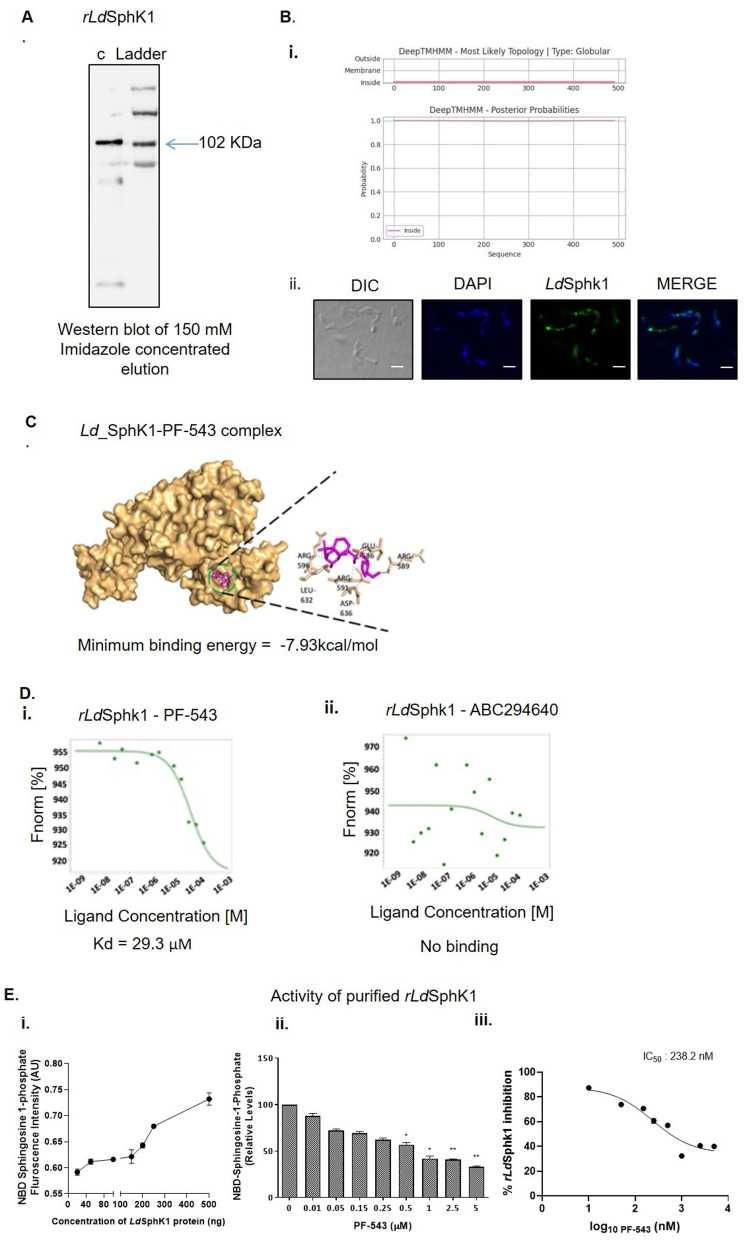
Purification, subcellular localization, and functional characterization of recombinant *rLd*SphK1 in *Leishmania donovani* in the presence and absence of the SphK1 inhibitor PF-543. (A) Concentrated *rLd*SphK1 protein. Gel image showing a purified band of 102 kDa recombinant LdSphK1 (B) The subcellular localization of recombinant *Ld*SphK1 was investigated in promastigotes. (i) DeepTMHMM results predicted globular location with no transmembrane domains with a probability of 1. (ii) Panel A: DIC at 60X. Panel B: DAPI. Panel C: anti-*Ld*SphK1 antibody detected using FITC (green)-conjugated secondary antibody. Panel D: merged micrographs (Scale bar - 20 µm). *Ld*SphK1 expression represented in the green channel. Co-localization shown as the merged image. (C) *In silico* docking of the *rLdSphK1* in presence of PF-543. *In-silico* ligand-substrate interaction was done using Autodock 1.5.7rc1 and Cygwin terminal. The *in silico* docking generated the complex of *rLdSphK1*-PF-543 and represented as surface model highlighted with residues involved in the interaction using Chimera, Ligplus, Discovery Studio v19.1.0, and PyMOL v2.3.2 software. (D) Biophysical interaction and functional characterization of the *rLdSphK1* (20 µM) in presence of PF-543. MST analysis confirms the interaction of *rLdSphK1* with PF-543 and ABC294640. Dose-response curves of *rLdSphK1*–PF-543 showed K_D_ of 29.3 µM and no interaction between *rLdSphK1* and ABC294640. (E) Analysis of functional characterization of the *Ld*SphK1 recombinant protein. (i) Time kinetics of activity of purified *rLd*SphK1 and NBD-S1P-based fluorometric assay showed increase in NBD-SIP levels under the influence of varying concentrations (20ng - 500ng) of *rLd*SphK1 protein. (ii) The inset shows inhibition of NBD-SIP levels in the presence of different concentrations of PF-543 (0.01µM - 5µM) using 200ng of recombinant *Ld*SphK1 in view of sharp increase of NBD-S1P levels at 200ng of *rLd*SphK1 protein observed in previous experiment.

*Ld*Sphk1 (1–2808 bp Nucleotide sequence, **S3A Fig**) was successfully cloned in pET-28 a(+) vector with N-terminal 6x-Histidine-tag **(****S3B Fig****)** and recombinant protein was purified using affinity based purification method **(****S3C Fig**). For this, we went for expression and purification of the recombinant *Ld*SphK1 (*rLd*SphK1) protein and observed a single band corresponding to ~102 kDa on SDS PAGE **(Fig 2A)** corresponding to *Leishmania* specific SphK1. This confirmed the availability of purified enzyme suitable for functional and structural analyses.

To investigate the cellular location of *rLd*SphK1, a DeepTMHMM-based *in silico* topology analysis was performed. The results predicted, with high confidence that *Ld*SphK1 is a globular, intracellular protein with zero transmembrane domains, confirming its soluble, intracellular nature. This finding aligns with our immunofluorescence data and further supports previous reports [15] describing cytoplasmic and membrane-associated localization of SphK1 in eukaryotic systems. Consistent with these observations, subcellular localization of *rLd*SphK1 was investigated in *L. donovani* Bob cells using confocal microscopy to validate the in-house–generated anti-*rLd*SphK1 antibody. Immunostaining of *L. donovani* revealed predominant cytoplasmic and peripheral membranous localization of *rLd*SphK1 **(Fig 2Bii)**, in agreement with its predicted non-integral membrane nature.

Owing to the fact of the recent development in structural biology, notably with the awarding of the 2024 Nobel Prize in Chemistry to Demis Hassabis and John Jumper for their pioneering work on the AlphaFold model, our analysis also incorporated AlphaFold-predicted structures for comparative modelling where-in both AlphaFold and a 3D homology model of *Ld*SphK1 were generated. The 3D homology model of *Ld*SphK1 was validated by its stereochemical statistics, featuring a MolProbity score of 1.74 and 81.99% of residues in favoured Ramachandran regions, which were superior to the AlphaFold model **(****S4****A and** **S4B Fig****).** Crucially, we assessed the local quality of the binding pocket predicted by both models. While the key interacting residues from our docking simulation (e.g., ARG589, GLU586, etc.) were confirmed to be in Ramachandran-favoured regions in our homology model, the overall environment of the binding site is of significantly higher quality. The near-perfect clash score of 0.29 in our model compared to 2.07 for the AlphaFold model indicates a much more physically realistic and geometrically accurate binding pocket, which is essential for reliable docking **(****S4****A and** **S4B Fig**). Given that our homology model demonstrates a superior MolProbity score and a virtually clash-free binding site, we concluded it provides a more reliable foundation for our docking analysis.

With a validated structural model, docking analysis of the *Ld*SphK1 protein with the inhibitor PF-543 was performed. The optimal conformations of the docked compounds were chosen based on their minimum free binding energy to the binding domain. Upon analysis of this pocket, it was discovered that there is a single hydrogen bond between residue ARG589 and ligand, with a distance of 2.03 Å. In addition, GLU586, ARG591, ARG596, LEU632, and ASP636 established hydrophobic contacts within the pocket. The binding of PF-543 to the protein is facilitated by these interactions, which result in a minimum binding energy of -7.93 kcal/mol **(Fig 2C)**. The analysis of these results indicates that PF-543 creates a stable structure when bound to the *Ld*SphK1 binding pocket.

Our analysis revealed a significant and exploitable difference between the parasite and human enzymes. The crystal structure shows that PF-543 is anchored in the human SphK1 active site primarily via hydrogen bonds to ASP264 [55]. Our docking model for *Ld*SphK1, however, predicts that the key anchoring interaction is a hydrogen bond with Arg589 **(Fig 2C)**. Notably, the binding mode of PF-543 in *Ld*SphK1 is distinct from human SphK1, further establishing the specificity of this interaction.

To validate the biophysical interaction between purified *rLd*SphK1 and PF-543 and analyze the binding and kinetics, Microscale Thermophoresis (MST) was performed in NanoTemper Monolith NT.115 instrument. Herein, labelled *rLdSphK1* (20 µM) was titrated against varying concentrations of PF-543 (500nM, 1µM, 2.5µM, 5µM, 10µM, 20µM). The dose response analysis for *rLdSphK1*-PF-543 binding revealed dissociation constant, K_D_ to be 29.3 µM, suggesting measurable and specific binding of *rLdSphK1* with PF-543 **(Fig 2D).** Additionally, we also evaluated interaction between *rLdSphK1* and ABC294640 as a negative experimental control where no such binding between the two could be detected, underscoring the specificity of PF-543 for *rLd*SphK1.

To evaluate the functional competence of *rLd*SphK1, the time course kinetics for *rLdSphK1* was done using NBD-Sphingosine based fluorometric assay for detection of NBD-S1P levels Across the tested enzyme range (20ng to 500ng), a pronounced increase in NBD-S1P levels was observed as *rLd*SphK1 concentration increased from 200ng to 500ng **(Fig 2Ei),** following which saturation in activity was detected. In addition, the inhibition of *rLd*SphK1 catalytic activity was also evaluated in a dose dependent manner with PF-543’s concentration ranging from 0.01µM to 5µM. The results represented IC₅₀ close to 500nM (used for experimentation) and a maximum inhibition at 5µM of PF-543, while the protein concentration was kept constant at 200ng **(Fig 2Eii),** further corroborating effective target engagement.

To detect Km and Vmax values, varying concentration of NBD Sphingosine (2–10µM) was taken along with 200ng of *rLd*SphK1. The results confirmed 10.48μM and 10.91μM/sec as Km and Vmax respectively, according to Lineweaver-Burk plots, while D-erythro-sphingosine is used as the substrate for *Ld*SphK1 **(****S3D Fig****)**, confirming robust enzyme activity under these conditions. Our *in silico* docking, MST, and enzymatic inhibition data are internally consistent, all supporting specific binding to parasite SphK1.

### 3. Expression and Purification of the recombinant human SphK1 (*rhSphK1*) protein and its Functional Characterization in presence and absence of SphK1 inhibitor- PF-543

To evaluate the similarities and evolutionary divergence between *Leishmania* and human SphK1, we first performed amino acid sequence alignment of *Ld*_SphK1 and human_SphK1 using the MEGAX program (version 10.2.2). The analysis revealed considerable agreement, with 26% sequence identity over 58% query coverage **(****S5A Fig****)**. A phylogenetic tree was also constructed to assess the evolutionary relationship between *Leishmania* SphK1 and its orthologs. The resulting tree shows a clear separation between the kinetoplastid and mammalian enzymes, which form distinct, highly supported clades. Within the kinetoplastids, all *Leishmania* species form a monophyletic group, demonstrating the conservation of this enzyme across the genus and its divergence from the host enzymes **(****S5B Fig****)**. Further, we conducted 3D structural alignment of the two protein structures with Pymol software. We observed substantial alignment between the two proteins, with an RMSD of 0.585Å **(****S5C Fig****),** demonstrating notable conservation of key structural features despite low overall sequence identity with notable correspondence between the alpha helices and beta sheets of the human (blue) and *Leishmania* (yellow) DAGKc domains **(****S5C Fig****).**

**Fig 3:**
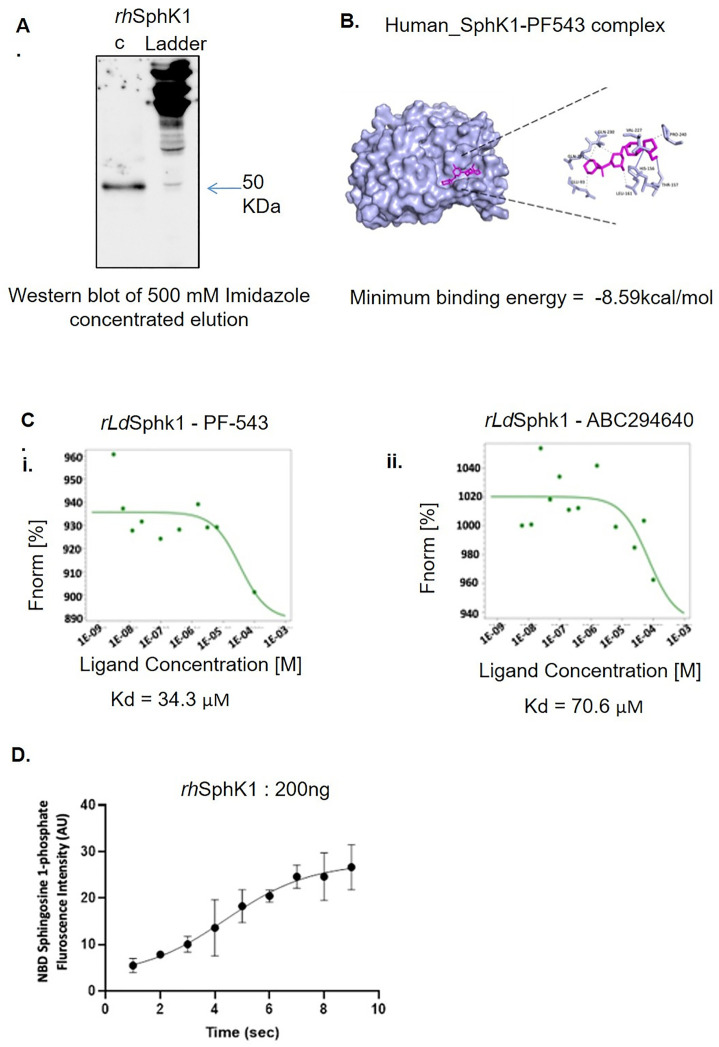
Purification, Molecular Docking, and Functional Characterization of Recombinant rhSphK1 in the presence of inhibitors. (A) Purification of Recombinant *rhSphK1* from Supernatant. Gel image showing a concentrated *rhSphK1* protein band of 50kDa. (B) *In silico* docking of the *rhumanSphK1* in presence of PF-543. *In silico* ligand-substrate interaction was done using Autodock 1.5.7rc1 and Cygwin terminal. The *in silico* bound complex of *rhSphK1*-PF-543 was represented as surface model highlighted with residues involved in the interaction using Chimera, Ligplus, Discovery Studio v19.1.0, and PyMOL v2.3.2 software. The list of residues involved in hydrophobic interaction and hydrogen bond formation were highlighted in the model. (C) Functional characterization of the *rhSphK1* in presence of PF-543. MST analysis confirms the biophysical interaction of *rhSphK1* with (i) PF-543 and (ii) ABC294640, which was used as a control. Dose-response curves of PF-543 (20µM) and ABC294640 showed K_D_ of 34.3µM and 70.6µM respectively. (D) Analysis of catalytic activity of the *rhSphK1* using NBD-S1P-based fluorometric assay. Time kinetics of activity of purified *rhSphK1* demonstrated increase in NBD-SIP levels in presence of 200ng of *rhSphK1* protein.

Building on this, the *human*Sphk1 gene was successfully cloned in pET-28 a(+) expression vector, and the recombinant plasmid was transformed into *E. coli* strain BL21 (ʎDE3) gold**.** Later, we went for expression and purification of the recombinant *human*SphK1 (*rhuman*SphK1) protein **(****S6A Fig****).** A single band corresponding to ~50 kDa was observed on SDS PAGE **(Fig 3A**) corresponding to recombinant human specific SphK1.

To elucidate the potential for pharmacological targeting of hSphK1, *in-silico* docking studies were performed between the protein and the SphK1 inhibitor PF-543. Upon examination of this pocket, it was discovered that it contains two residues, namely HIS156 and THR157, which are linked together by hydrogen bonds. The hydrogen link between them has a distance of 3.44 Å, while the other hydrogen bond has a distance of 2.53 Å. Similarly, GLU93, LEU161, VAL227, GLN230, GLN231, and PRO240 established hydrophobic interactions within the pocket. The binding of PF-543 to the protein is facilitated by these interactions, resulting in a minimum binding energy of -8.59 kcal/mol **(Fig 3B).** The analysis of these results indicates that PF-543 creates a stable structure when bound to the hSphK1 binding pocket. These data inferred that *rhSphK1* and PF-543 are possible interacting partners having favourable binding energy.

To corroborate these findings experimentally, biophysical binding assays (MST) was performed in NanoTemper Monolith NT.115 instrument. Herein, labeled *rhSphK1* (20 µM) was titrated against varying concentrations of PF-543 and the SphK2 inhibitor ABC294640 as a control. Dose–response analysis revealed dissociation constants (K_D_) of 34.3 µM for *rh*SphK1–PF-543 and 70.6 µM for *rh*SphK1–ABC294640, confirming specific and preferential binding **(Fig 3C).** Here, *rh*SphK1 MST experiments were performed under identical buffer and labeling conditions as *rLd*SphK1 to enable direct comparison, not to re-define canonical affinity. We clearly state that experimental MST-derived K_D_ values are method- and context-dependent and do not replace previously published enzymatic IC_50_ values (reported in nanomolar range).

Functional activity of the recombinant protein was assessed using NBD-sphingosine–based fluorometric detection of NBD-S1P formation. Kinetic analysis represented a steep increment in generation of NBD-S1P level over time with 200 ng *rhSphK1* **(Fig 3D)** [45, as standardized for *rLd*SphK1], following which saturation in activity was detected. To detect Km and Vmax values, varying concentration of NBD Sphingosine (2–10 µM) was taken along with 200 ng of *rh*SphK1. The results confirmed 9.56μM and 9.13μM/sec as Km and Vmax respectively with D-erythro-sphingosine is used as the substrate for *rhSphK1* **(****S6B Fig****).**

Together, these results demonstrate the successful expression and purification of functional recombinant human SphK1, demonstrate its evolutionary and structural conservation with the *Leishmania* enzyme, and confirm robust inhibitor binding and enzymatic activity under defined experimental conditions.

### 4. Effect of PF-543 on cytotoxicity and infectivity of *L. donovani* SphK1 overexpressor *(Ld*SphKa) promastigotes

**Fig 4:**
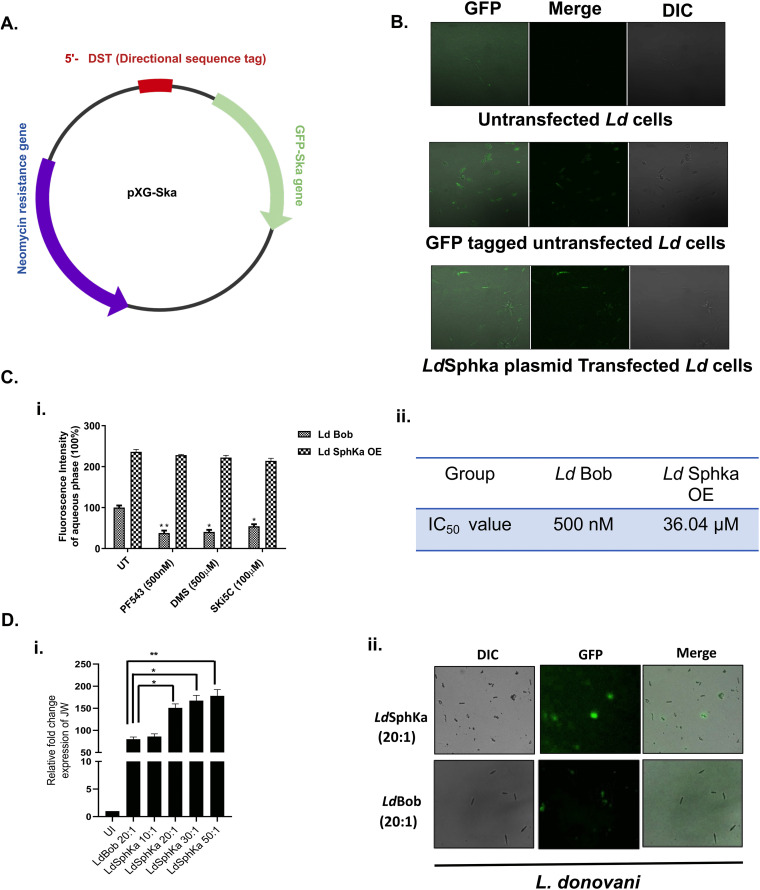
Effect of PF-543 on cytotoxicity and infectivity of *L. donovani* SphK1 overexpressor promastigote cell line. (A) Vector map showing the construct designed for pXG plasmid containing SphKa gene and GFP for the overexpression of the gene through transfection in *Ld*Bob cells. (B) Confocal microscopy images confirming successful integration of the plasmid. Integration was verified by the presence of GFP fluorescence signal in the transfected parasites. (C) (i) Estimation of LdSphK-1-mediated generation of NBD-SIP levels against PF-543 in *L. donovani* SphKa promastigotes. *L. donovani* bob promastigotes were cultured in 25 cm^2^ flasks followed by treatment with SphK1 inhibitor; PF-543 for 48h. Inhibition of SphK1 using PF-543 had no significant effect on S1P levels. SphK inhibitor treated SphKa promastigotes were resuspended in buffer containing fatty acid-free BSA (0.1% (w/v)) followed by resuspension in buffer containing BSA (1% (w/v)) and incubated with NBD-sphingosine (10μM) for 45 min at 37°C. Promastigotes incorporate NBD-sphingosine (NBD-Sph), which are phosphorylated by Sphingosine Kinase (SphK1) to NBD-S1P. Bar graph depicts ELISA-based S1P quantification in SphKa promastigotes treated with PF-543 using *Ld*Bob as control. (ii) Comparative analysis of the half maximal inhibitory concentration of PF543 against the wild type *Ld*Bob and *Ld*Sphka OE line. (D) Effect of SphK1 inhibitor; PF-543 in *Leishmania* overexpressor *spp*. (i) Effect of *L. donovani* SphK1 overexpression on intracellular parasite clearance. THP-1 macrophages were cultured in six-well plates in the presence or absence of *L. donovani* (*Ld*Bob) infection (MOI, 20:1) for 6h. Further, THP-1 cells were infected with *L. donovani* SphK1 overexpressor promastigotes at various MOIs (10:1, 20:1, 30:1, 50:1). Infected THP-1 were washed to remove non-internalized parasites. Total RNA was enriched using TRIzol and the resulting cDNA was subjected to real-time PCR analysis using primers specific for infectivity (JW) genes in infected macrophages. RNU6A was used as a housekeeping gene. The results are expressed as fold-change of uninfected THP-1 cells. Statistical significance was quantified using the unpaired t-test. The data is a representation of mean ± SD from three independent experiments *, p < 0.05; ***, p < 0.001. (ii) GFP-based confirmation of SphK1 overexpression in *L. donovani* promastigotes. Detection of the presence of GFP encoded SphKa and *Bob* plasmids in *Leishmania donovani* and *Leishmania* overexpressor parasites. *L. donovani bob* and *L. donovani* overexpressor promastigotes were cultured in 25 cm^2^ flasks followed by confocal microscopy.

To verify transient genetic manipulation, *Leishmania donovani* promastigotes were successfully transfected with the pXG-GFP-SphKa construct (**Fig 4A**), in which the SphK1 gene was fused at the 3′ end of GFP to enable fusion protein detection. Confocal microscopy revealed a distinct population of GFP-positive parasites, confirming transgene expression, with a subset of GFP-negative cells and untransfected controls included to validate specificity **(Fig 4B).** This robust GFP signal established the basis for subsequent functional studies.

The functional activity of *Leishmania*-specific SphK1 in over-expressor promastigotes (*Ld*SphKa) was confirmed using fluorometric quantification of NBD-tagged sphingosine conversion. Treatment with generic SphK inhibitors (PF-543, DMS, SKI-5C) resulted in a marked reduction in NBD-S1P fluorescence intensity compared to untreated *Ld*Bob promastigotes, demonstrating effective inhibition of SphK catalytic activity across all tested compounds and doses (500nM PF-543; 100 µM SKI-5C; 500 µM DMS). Notably, *Ld*SphKa-overexpressing parasites displayed elevated baseline NBD-S1P levels relative to *Ld*Bob controls, directly supporting both the overexpression and functional activity of the enzyme **(Fig 4Ci).** These data establish that *Ld*SphK1 overexpression leads to enhanced SphK1 enzymatic output in *Leishmania donovani*.

Further dose-response inhibition studies demonstrated that each inhibitor showed comparable suppression of SphK1 activity in *Ld*SphKa lines. These findings are mechanistically explained by the greater absolute SphK1 expression in *Ld*SphKa compared to *Ld*Bob, resulting in negligible changes in activity for *Ld*Bob at inhibitor concentrations used. Thus, these data confirm both the inhibitor specificity and the physiological relevance of SphK1 overexpression in mediating enzymatic output.

Given the robust inhibition by PF-543, its potency was analyzed in greater detail. MTT cytotoxicity assays revealed that PF-543 elicited an IC₅₀ of 36.04µM against *Ld*SphKa promastigotes (Fig 4C ii), which was higher than observed for wild-type *L. donovani* promastigotes, indicating a degree of resistance conferred by SphK1 overexpression, likely through modulation of downstream lipid signaling pathways.

The functional consequences of SphK1 overexpression on parasite infectivity were assessed through infection of THP-1 cells at multiple MOIs (10:1, 20:1, 30:1, 50:1), with intracellular amastigote loads quantified by RT-PCR 48h post-infection. *Ld*SphKa-infected THP-1 cells exhibited pronounced infection rates (approximately 86% at MOI 10:1 versus ~80% at MOI 20:1 for *Ld*Bob controls), with higher MOIs further supporting a rapid-growth, high-infectivity phenotype (Fig 4D i). Strong GFP fluorescence in *Ld*SphKa promastigotes and absence in *Ld*Bob controls (Fig 4D ii) further confirmed correct genetic targeting and specificity.

### 5. Decrease in *Leishmania* glutathione levels mediates ceramide-induced parasite death

Ceramide, a central sphingolipid metabolite, lies upstream of SphK1 in the sphingolipid biosynthetic pathway and is antagonized by Sphingosine-1-phosphate (S1P), an established pro-survival lipid mediator. During *Leishmania* infection, elevated ceramide production disrupts host membrane lipid rafts, lowers cholesterol, and impairs CD40-mediated signaling, ultimately weakening ERK1/2 activation and antigen presentation, contributing to parasite survival and disease progression [56].

**Fig 5:**
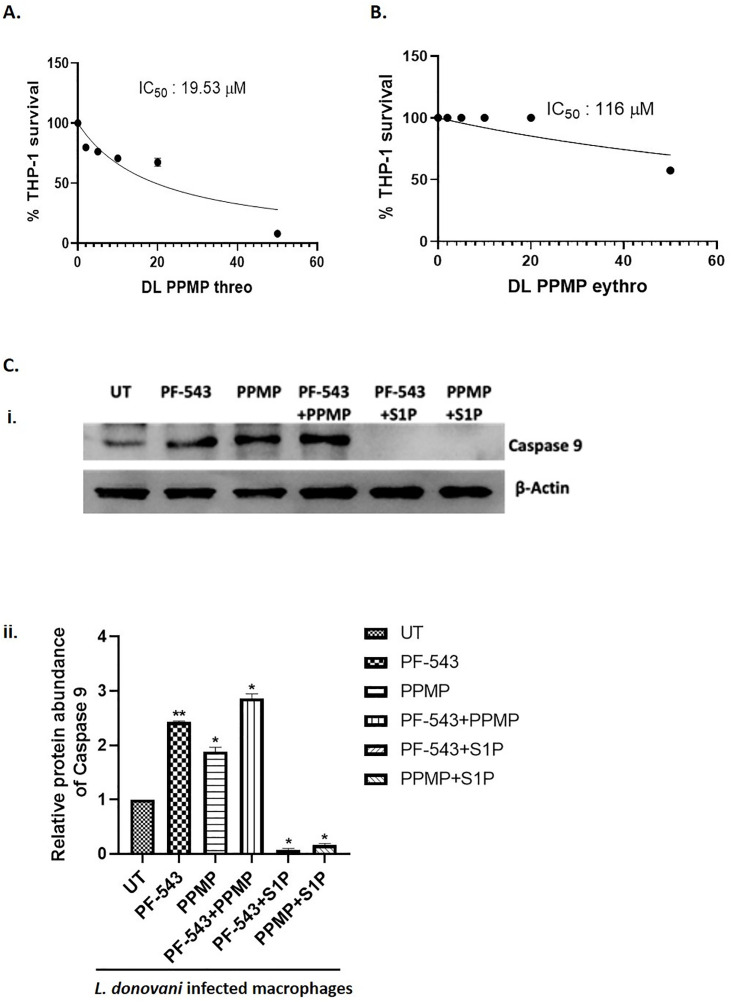
Assessment of DL-PPMP cytotoxicity and the effect of PF-543 on Caspase-9 activation in Leishmania donovani-infected macrophages. (A) Determination of *in vitro* cytotoxicty of DL PPMP threo and DL PPMP erythro. DLPPMP threo and DLPPMP erythro at varying concentrations (2µM, 5µM, 10µM, 20µM and 50µM) were incubated with THP-1 cells seeded 6 × 10^3^ in a 96-well plate for 48h. The cytotoxicity was analysed using colorimetric estimation of formazan crystals formed by the conversion of MTT by live cells. IC_50_ determined by plotting the non-regression linear curve was 19.53µM and 116µM for DLPPMP threo and erythro respectively. (B) Estimation of Caspase 9 levels upon PF-543 treatment in Leishmania infected macrophages. (i) THP-1 macrophages were cultured in six-well plates in the presence of *L. donovani* infection (MOI, 20:1) for 6h. Infected THP-1 were washed to remove non-internalized parasites and treated with PF-543 (20μM) or DL-threo-PPMP; glucosylceramide synthase inhibitor (20μM) or SIP (10μM) for next 48h. Caspase-9 levels were analysed using cell lysates for Western blot. (ii) Band intensities were quantified by ImageJ software and were plotted in GraphPad Prism. Data from one of three experiments is shown.

To interrogate the role of ceramide in parasite-induced cytotoxicity, the ceramide analog DL-threo-PPMP, a known inhibitor of glucosylceramide synthase, was employed. Dose-response experiments revealed that exogenous DL-threo-PPMP (20μM) exerts a potent cytotoxic effect on THP-1 macrophages, as indicated by reduced cell survival (IC₅₀ = 19.53μM, **Fig 5A**). In contrast, the isomeric control DL-erythro-PPMP displayed significantly lower potency (IC₅₀ = 116μM, **Fig 5B**), validating the isomer-specific action of DL-threo-PPMP and supporting its selection for subsequent studies.

Next, THP-1 cells infected with *L. donovani* (MOI 20:1) were exposed to DL-threo-PPMP. Subsequent biochemical analysis demonstrated that PPMP treatment led to a marked decrease in the GSH/GSSG ratio (indicative of oxidative stress, **S7 Fig**) and a pronounced induction of apoptosis, evidenced by elevated Caspase 9 expression **(Fig 5C i).** These findings confirm that ceramide accumulation drives parasite-induced host cell death through glutathione depletion and apoptotic signalling.

To dissect the functional interplay between ceramide and S1P, infected macrophages were co-treated with both DL-threo-PPMP and S1P (10μM, a non-toxic concentration for parasites). Remarkably, S1P supplementation abolished ceramide-induced cytotoxicity, restoring GSH/GSSG ratios and suppressing Caspase 9 activation (**Fig 5Cii).** Correlation analysis further revealed a strong negative association between glutathione levels and apoptotic signalling (Pearson r = –0.78, p = 0.05), supporting a direct mechanistic link.

Collectively, these data demonstrate that S1P acts as a downstream physiological antagonist to ceramide-induced stress and apoptosis in *Leishmania*-infected macrophages, highlighting the pivotal role of glutathione balance and the ceramide-SphK1-S1P axis in regulating host-parasite interactions and cell fate decisions.

### 6. Elucidation of inflammatory responses and infectivity post-PF-543 treatment in *Leishmania* infected macrophages

**Fig 6:**
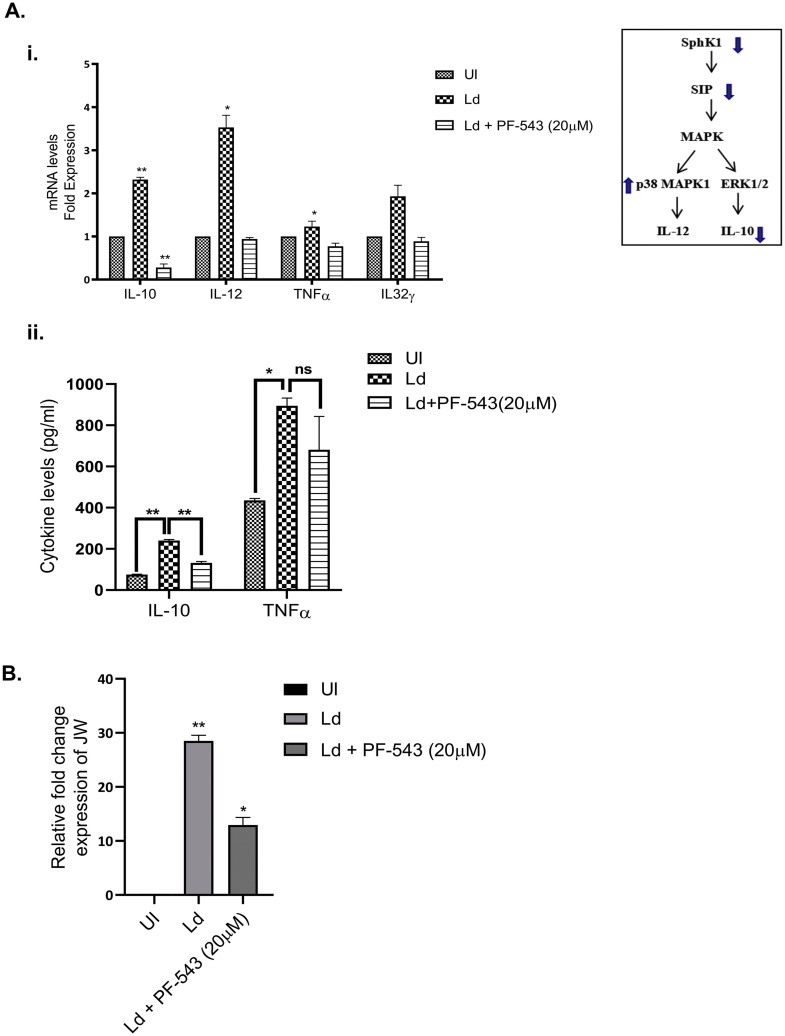
(A(i), B) Effect of PF-543 on the expression of inflammatory molecules and clearance of intracellular parasite load. THP-1 macrophages were cultured in six-well plates in the presence or absence of *L. donovani* infection (MOI, 20:1) for 6h. Infected THP-1 were washed to remove non-internalized parasites and treated with PF-543 for next 48h. Total RNA was enriched using TRIzol and the resulting cDNA was subjected to real-time PCR analysis using primers specific for inflammatory (IL32γ, IL-12, IL-10 and TNF-α) and infectivity (JW) genes in PF-543 treated and untreated infected macrophages. RNU6A was used as a housekeeping gene. The results are expressed as fold-change of uninfected THP-1 cells. Statistical significance was quantified using the unpaired t-test. The data is a representation of mean ± SD from three independent experiments *, p < 0.05; ***, p < 0.001. (A (ii)) Graph represents cytokine levels measured by sandwich ELISA. Statistical significance was quantified using the unpaired t-test with Welch’s correction, in LPS-stimulated, *Ld*-infected and PF-543 treated macrophages.

Cytokines play a crucial role in mediating T-cell responses and host defense mechanisms in macrophages. LPS potently activates macrophages and cytokine signalling by TLR4 stimulation, while *Leishmania* parasites suppress LPS-induced host inflammatory cytokine responses [57]. To probe the effect of SphK1 pathway inhibition on host inflammatory balance, we assessed cytokine profiles following PF-543 treatment of LPS-stimulated, *Leishmania*-infected macrophages. We found that PF-543 post-treatment in infected macrophages reduced the levels of anti-inflammatory cytokine *IL-10* (~80%) as confirmed by RT-PCR and ELISA while the level of pro-inflammatory cytokines *IL32γ*, *IL-12* and *TNF-α* remain unaltered **(Fig 6Ai, and** **6Aii).** We further evaluated the parasite load in presence of PF-543 using qRT-PCR based on estimation of JW (kDNA) mRNA levels in PF-543 treated and untreated infected cells. The results suggested PF-543 post-treatment leads to decrease (_~_50%) in parasite load in infected macrophages **(Fig 6B),** thereby indicating the role of PF-543 in reducing the parasite survival.

Together, these findings highlight the key role of the SphK1/S1P axis in regulating anti-inflammatory cytokine production and parasite persistence within macrophages, and demonstrate that pharmacological inhibition with PF-543 has both immunomodulatory and anti-parasitic effects in the context of *Leishmania* infection.

### 7. Evaluation of antileishmanial activity of PF-543 and Amphotericin B combined formulation against *L. donovani* promastigotes and intracellular amastigotes

**Fig 7:**
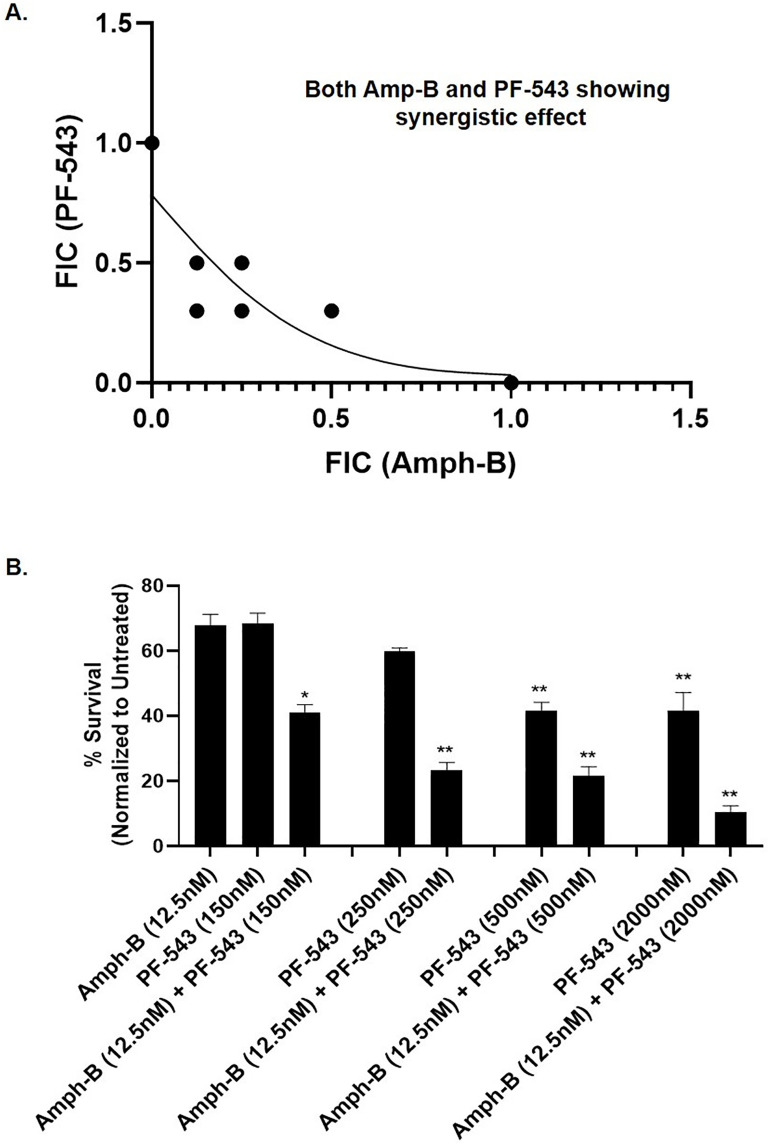
(A-B). Evaluation of PF-543 and Amphotericin B formulation on cytotoxicity or metabolic viability of *Leishmania* promastigotes and intracellular amastigotes. (A) To determine the combinatorial impact of PF-543 and Amphotericin B formulation, promastigotes of *L. donovani* were seeded in 96-well plates (3 × 10^6^ parasite/mL) in exponential growth phase at increasing concentrations (12.5nM–500nM) of Amphotericin B and (150nM- 2000nM) of PF-543 compounds and maintained at 26°C. After 48h of incubation, the IC_50_ was determined by MTT assay. The absorbance was measured in a MultiskanEX photometric plate reader for microplates at 540 nm. Data were obtained from three independent experiments performed in triplicate. The fractional inhibitory concentration index (FIC) was evaluated to determine the synergistic activity of lead compounds; PF-543 with Amph-B. Finally, FIC < 0.5 represented strong synergistic activity between two drugs. While FIC > 4 is was considered as the antagonistic activity. FIC values were obtained from three independent experiments. **(B)** For validating the antileishmanial activity by PF-543 and Amphotericin B formulation on intracellular amastigotes, qPCR-based analysis was performed for all the treatments. For the same, THP-1 cells were seeded at a concentration of 8 × 10^5^ cells/mL in 96-well plates and incubated for 24h with PMA (10ng/mL) supplemented with RPMI 1640. Following 24h priming with PMA, the culture medium was removed, and the cells were infected with *L. donovani* followed by treatment with the formulations having concentrations kept similar as used previously for promastigotes at 37°C and 5% CO_2_. Total RNA was extracted, and the resulting cDNA was subjected to real-time PCR analysis using primers specific for kinetoplast minicircle DNA (kDNA)/JW of *L. donovani*. This primer does not match to human genome, thus directly indicates the intracellular parasite load against the treatments. Data analysis was performed using the 2^-ΔΔCT^method.Values are mean ± S.D (n = 3). The results are representative of three independent experiments performed in triplicates.

Amphotericin B (Amph-B) remains a cornerstone of visceral leishmaniasis treatment [58] due to its ergosterol-binding mechanism, which disrupts protozoan membrane integrity and induces cell death [59]. To explore the therapeutic potential of combining PF-543, a SphK1 inhibitor, with Amph-B, *in vitro* anti-leishmanial assays were conducted in *L. donovani* promastigotes using the MTT assay. The *in-vitro* synergistic activity of PF-543 along with Amphotericin B was represented by an isobologram. Upon analysis of antileishmanial activity, Amphotericin B showed an IC_50_ of 30nM and PF-543 showed IC_50_ of 500nM alone (as already shown in **Fig 1B**) however upon administering both PF-543 and Amphotericin B in combination, synergistic impact was prominent, and the IC_50_ was observed to be ~ 12.5nM and 150nM for Amphotericin B and PF-543 respectively, as depicted by the isobologram micrographs in promastigotes **(Fig 7A).**

For validating the anti-leishmanial activity by PF-543 and Amphotericin B formulation on intracellular amastigotes, qPCR-based analysis was performed for all the treatments. Here-in the synergistic treatment of Amphotericin B (12.5nM) and PF-543 (150nM) formulation to intracellular amastigotes reduced the parasite growth by 60%. Whereas, treatment with PF-543 or Amphotericin B alone decreased the parasite burden by 30%. Further reduction of 40% and 60% in parasite growth was observed with usage of higher dosage of PF-543 alone (250nM, 500nM and 2000nM), while these doses in combination with Amphotericin B (12.5nM + 150nM, 12.5nM + 250nM, 12.5nM + 500nM, 12.5nM + 2000nM) showed higher reduction of parasite growth to ~80–90% **(Fig 7B).** These data clearly suggested that PF-543 along with Amphotericin B can be a promising potent formulation to be tested.

### 8. Elucidation of inflammatory responses and infectivity post-PF-543 treatment in *Leishmania*-infected Swiss mice

PF-543 is a potent and selective sphingosine kinase 1 (SphK1) inhibitor that has shown notable anti-cancer activity in preclinical studies by blocking SphK1-mediated signaling pathways, leading to the induction of apoptosis and growth inhibition in various cancer cell lines. It is also reported to exhibit low systemic toxicity at experimentally determined doses in mice (typical half-life in mice is approximately 1–1.2 hours). Amphotericin B, by contrast, is a well-established anti-leishmanial agent approved for clinical use. Both were selected for the present study due to established safety and efficacy profiles *in vivo.*

To investigate the anti-leishmanial efficacy and immunomodulatory impact of PF-543 in a murine model, Swiss mice were infected with *L. donovani* and subsequently treated intraperitoneally with PF-543 (10 mg/kg), Amphotericin B (2 mg/kg), or a combination regimen (PF-543 2 mg/kg + Amph-B 0.4 mg/kg) for three consecutive days. Dose selection was based on prior reports and pilot tolerability studies, ensuring all regimens were well tolerated without adverse effects or significant weight loss. These criteria align with established clinical protocols for Amphotericin B and emerging preclinical data for SphK1 inhibitors.

To evaluate the parasitaemia and expression of inflammatory markers in infected mice upon drug administration, spleen was harvested from each group of mice and qRT-PCR analysis was performed using primers specific for inflammatory markers; *mTNF-α* and *mIL-10* **(****S1 Table****)** and parasite specific kinetoplast minicircle gene; *JW* in PF-543 and Amph-B treated infected mice in respect to the untreated Swiss mice.

**Fig 8:**
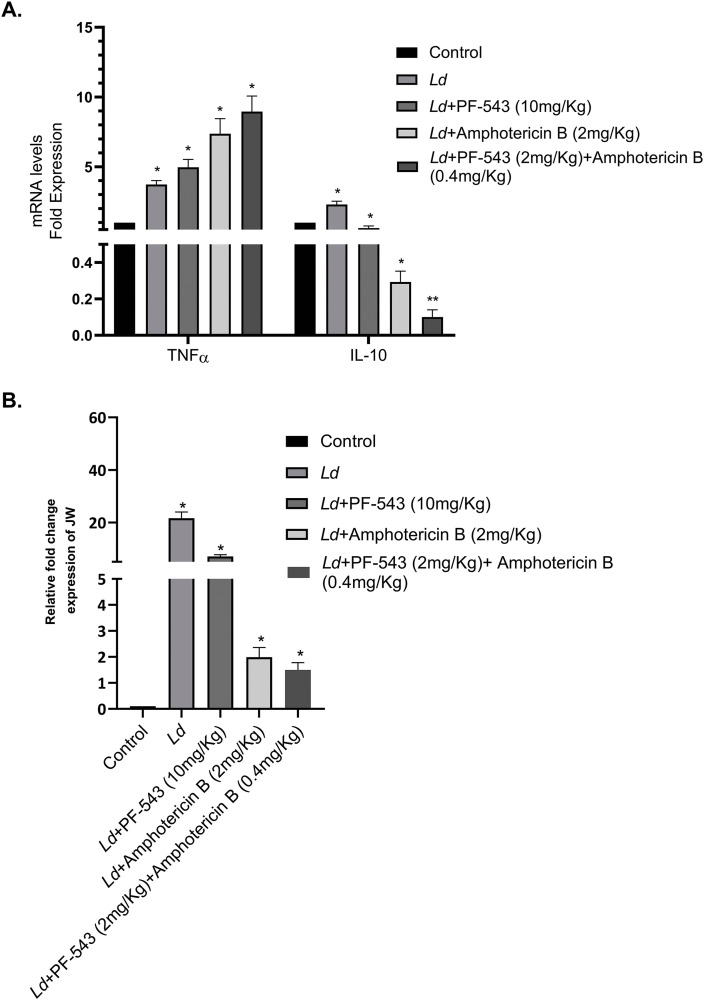
Expression of JW and inflammatory markers upon PF-543 treatment in *Leishmania-infected* Swiss mice. Evaluation of the **(A)** immunomodulatory potential and **(B)** infectivity of PF-543 and Amph-B in *Leishmania*-infected Swiss mice was performed. 10mg/Kg of PF-543 and 2mg/Kg of Amphotericin B alone were given for individual treatment in *Leishmania* infected mice. For constituting the synergistic effect of two drug partners; 2mg/Kg (1mM) of PF-543 and 0.4mg/Kg (0.12µM) of Amphotericin B were prepared in 1XPBS and given together to a mouse weighing 30gm. To evaluate the parasitaemia and expression of inflammatory markers in infected mice upon drug administration, spleen was harvested from each group of mice and qRT-PCR analysis was performed using primers specific for inflammatory markers *IL-10 and TNFα* and parasite specific kinetoplast minicircle gene, *JW* in PF-543 and Amph-B treated infected mice in respect to the untreated Swiss mice. RNU6A (RNA, U6 small nuclear 1; THP-1 cells) was used as a housekeeping gene. The results were expressed as normalized fold-change of respective control. Statistical significance was quantified using the unpaired t-test. The data is a representation of mean ± SD from three independent experiments *, p < 0.05; ***, p < 0.001.

In concordance with the published studies, here-in, upon analysis, treatments with either Amphotericin B or PF-543 alone in infected mice resulted in a significant decrease in *IL-10* expression (~60%), whereas increased *TNF-α* expression was observed (1.5-2 folds) (**Fig 8A).** Further, this effect on inflammatory markers was more pronounced upon the synergistic treatment of Amphotericin B and PF-543 formulation to *Leishmania* infected Swiss mice (~90% decrease in *IL-10* & 3 folds increase in *TNF-α*) (**Fig 8A).** Amplification of RNU6AP (RNA, U6 small nuclear 1; THP-1 cells) was used as an internal control for normalization.

Most importantly, parasite load was also observed to be reduced upon treatment with PF-543 or Amphotericin B alone in infected mice (**Fig 8B**) where ~33% and ~80% reduction in parasite growth was observed respectively. Further, this down-regulation in parasite burden was drastically reduced to >90% (as demarcated by reduction in *JW* transcript) upon synergistic treatment of PF-543 and Amphotericin B formulation (**Fig 8B).**

Collectively, these *in vivo* results affirm that combination therapy with PF-543 and Amphotericin B leads to enhanced protective immune responses, substantially reduced regulatory cytokine (IL-10) levels, increased pro-inflammatory milieu (TNF-α), and synergistic clearance of *L. donovani* in an experimental mouse model. This highlights the promise of SphK1-targeted interventions as adjuncts in anti-leishmanial therapy.

## Discussion

The current study elucidates the role of Sphingosine Kinase 1 (SphK1) in the pathogenesis of *Leishmania donovani* and its potential as a therapeutic target. Our findings demonstrate that inhibition of SphK1 using PF-543, a potent and highly selective SphK1 inhibitor, leads to significant disruption in the parasite’s survival, proliferation, and host-parasite interactions, with moderate direct effects on parasite viability and a prominent contribution from host-mediated mechanisms. These effects correlate with emerging evidence that sphingolipid metabolism is a central node for controlling intracellular parasitism and immune evasion [60]. Furthermore, the combination of PF-543 with Amphotericin B shows promise as a synergistic treatment strategy, with enhanced efficacy against both promastigotes and intracellular amastigotes. This is consistent with recent reports where drug combinations have been shown to enhance treatment efficacy and reduce resistance [61].

The choice of PF-543 in this study is grounded in its superior selectivity, as it targets unique structural features within the SphK1 catalytic pocket not shared by SphK2, and exhibits sub-nanomolar binding affinity in biochemical and cellular assays [62]. In contrast, comparative agents such as DMS and SKI-5C show broader, less discriminating actions on sphingolipid metabolism. ABC294640, a SphK2-specific inhibitor, was included to provide definitive comparison of isoform-selective inhibition, further clarifying the unique contributions of SphK1 in both *Leishmania* and host macrophages [63].

Functional assays revealed that PF-543 exhibits limited cytotoxicity toward THP-1 macrophages, while exerting moderate inhibitory effects on promastigotes and intracellular amastigotes, with reduced potency against intracellular forms. This profile supports a context-dependent therapeutic window in which parasite growth is restricted without overt host cell toxicity, consistent with prior observations in infectious and cancer models [64]. The comparatively higher IC₅₀ values observed for axenic and intracellular amastigotes relative to promastigotes likely reflect restricted drug access, altered metabolism, and efflux within the intracellular niche. Notably, host cell IC₅₀ values remained higher than those required to reduce intracellular parasite burden, supporting a restricted but exploitable therapeutic window dominated by host-mediated parasite control rather than direct parasiticidal activity.

Molecular and cellular studies further illuminate this mechanism where-in recombinant *Ld*SphK1 was successfully cloned, purified, and confirmed to be active via NBD-sphingosine-based assays. PF-543 directly inhibited *rLd*SphK1 activity, lowering sphingosine-1-phosphate (S1P) production. Immunolocalization and topology analyses established cytoplasmic and membranous localization for *Ld*SphK1, matching known SphK1 cellular distribution in eukaryotes [15,61].

Docking and biophysical experiments indicated that PF-543 binds efficiently to key conserved residues (notably ARG589 and GLU586) in the *Leishmania* enzyme, supported by favourable kinetic binding properties (KD ~ 29µM for *rLd*SphK1; ~ 34µM for *rh*SphK1). Structural comparison revealed species-specific differences in anchor residues for PF-543 between *Leishmania* and humans, potentially guiding development of parasite-selective therapeutics [62]. *Ld*SphK1 represents an orthologue rather than a homologue of mammalian SphK1, sharing ~26% sequence identity across ~58% of the C-terminal region, including divergence within the catalytic pocket. *In silico* docking, MST, and enzymatic inhibition assays were internally consistent, all supporting specific binding to parasite SphK1. Under identical MST conditions, *rh*SphK1 also exhibited micromolar-range K_D_ values. These data are presented solely for comparative purposes and do not redefine canonical nanomolar enzymatic inhibition reported in prior biochemical studies.

According to the LeishGEM database, SphK1 is non-essential under promastigote culture conditions, consistent with the moderate phenotypes observed. However, non-essential genes may still represent viable pharmacological targets when overexpression increases resistance, inhibition compromises infectivity and intracellular survival and most importantly, dual targeting of parasite and host orthologues amplifies therapeutic outcome.

Overexpression studies in promastigotes further support SphK1’s centrality. The observed increase in IC₅₀ for PF-543 in *Ld*SphK1-overexpressing promastigotes suggests an important biological mechanism underlying partial resistance. Overexpression of SphK1 likely results in elevated basal levels of sphingosine-1-phosphate (S1P), thereby activating downstream pro-survival and proliferative signalling pathways such as AKT, NF-κB, and MAPK. Activation of these pathways can mitigate the cytotoxic effects of SphK1 inhibition and buffer the metabolic impact of PF-543. Additionally, the ceramide/S1P rheostat is known to influence susceptibility to cell death (as shown in our data), and increased SphK1 activity may shift this balance toward a more anti-apoptotic state, further contributing to drug resistance. Furthermore, the role of SphK1 in regulating MAPK signalling in *Leishmania*-infected macrophages has already been reported [65,66], supporting the involvement of SphK1 in modulating host–parasite signalling interactions. Thus, *Ld*SphK1-overexpressors retained partial resistance to PF-543 yet still showed substantial parasite load reductions [61]. This demonstrates that SphK1 not only promotes survival and infectivity but also modulates inherent drug susceptibility, likely by buffering against metabolic and signalling disruption.

At the cellular signalling level, results indicate that *Leishmania* infection shifts the ceramide/S1P balance towards a pro-survival phenotype. Inhibition of ceramide synthesis (with DL-threo-PPMP) reduces viability and increases stress/apoptotic signalling (elevated Caspase-9, reduced GSH/GSSG); S1P supplementation rescues these effects, reinforcing the hypothesis that SphK1-S1P acts as a critical determinant in host-parasite redox and survival signalling [61]. Here-in, we measured GSH/GSSG ratio primarily to evaluate the oxidative burden of the host THP-1 cells following infection and PPMP treatment. GSH is the predominant thiol-based redox buffer in mammalian cells, and its depletion is a well-established marker of host cell oxidative stress and apoptosis [67,68]. Since our study aimed to investigate the mechanism of host cell death induced by ceramide accumulation, GSH/GSSG served as a direct and relevant indicator of redox imbalance in the host.

Immunomodulation is a key outcome where-in SphK1 inhibition shifts infected macrophages away from an immunosuppressive, IL-10–dominated cytokine profile toward a pro-inflammatory, TNFα + /IL-12 + state. Such reprogramming is consistent with enhanced host control of intracellular infection, as shown here and in prior work [62].

Combination therapy with Amphotericin B, standard-of-care for leishmaniasis displayed strong *in vitro* and *in vivo* synergy. Co-treatment at sub-IC50 concentrations of each agent resulted in marked (>80%, and >90% in murine spleen) parasite reductions, outperforming monotherapy [61]. This synergy may reflect PF-543 induced membrane destabilization and S1P pathway blockade, sensitizing parasites to ergosterol-targeted damage.

Importantly, *in vivo* studies confirmed tolerability and efficacy of PF-543, Amphotericin B, and their combination. Treated mice exhibited robust reductions in parasite burden and favourable cytokine shifts, with no overt toxicity [60]. This validates SphK1 inhibition as a promising adjunct to conventional leishmanicidals.

**Fig 9:**
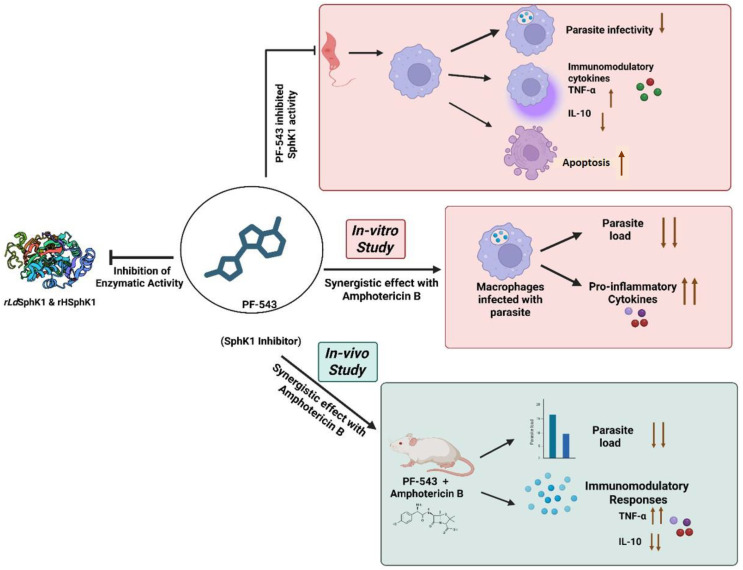
Schematic summary of the anti-leishmanial and immunomodulatory effects of SphK1 inhibition by PF-543. PF-543 inhibited recombinant *L. donovani* SphK1 activity, reduced parasite infectivity, and lowered amastigote burden (~40%), while shifting the cytokine profile toward a pro-inflammatory state (↑IL-12, ↑ TNF-α, ↓ IL-10). Inhibition of ceramide synthesis and rescue by S1P confirmed the role of the SphK1/S1P axis in parasite survival. Combination treatment with PF-543 and Amphotericin B showed synergistic efficacy (>90% parasite reduction) and enhanced host-protective immune responses, underscoring SphK1 as a promising therapeutic target in visceral leishmaniasis. Figure created in BioRender. Wali, A. (2025) https://BioRender.com/nit9sx0.

In summary, both recombinant *L. donovani* SphK1 (*rLd*SphK1) and human SphK1 (*rh*SphK1) exhibited robust enzymatic activity that was effectively inhibited by PF-543. In *L. donovani* promastigotes, PF-543 treatment significantly reduced SphK1 activity and parasite infectivity. Notably, PF-543 not only diminished parasite infectivity *in vitro* and decreased intracellular amastigote load by approximately 40%, but also skewed the host immune response toward a pro-inflammatory phenotype (↑IL-12, ↑ TNF-α, ↓ IL-10). Mechanistic analyses revealed that inhibition of ceramide synthesis and supplementation with sphingosine-1-phosphate (S1P) restored parasite viability, underscoring the role of the SphK1/S1P axis in parasite survival. Furthermore, combined treatment with PF-543 and Amphotericin B exerted synergistic anti-leishmanial activity both *in vitro* and *in vivo*, leading to over 90% reduction in parasite burden and enhanced immunomodulatory responses in infected macrophages and Swiss mice **(Fig 9,** [69]**)**. Collectively, this integrative study encompassing *in silico*, biochemical, cellular, and *in vivo* approaches provides compelling experimental evidence that targeting the SphK1/S1P signaling pathway represents a promising therapeutic strategy against visceral leishmaniasis. The findings highlight dual benefits through direct parasite killing and host immune modulation, advocating the translational potential of sphingolipid pathway inhibitors, especially in rational combination therapies, for leishmaniasis and other intracellular infections.

This study provides proof-of-concept evidence that simultaneous modulation of parasite and host SphK1 orthologues using PF-543 (already established as a clinical molecule for cancer, indicating its safety), can restrict *Leishmania* growth and survival. PF-543 functions as a dual-acting host–parasite modulator, exhibiting measurable parasite enzyme engagement alongside robust host-directed immunological effects. While not a parasite-selective nanomolar inhibitor, targeting the SphK1/S1P axis particularly in rational combination therapies represents a translationally relevant strategy for visceral leishmaniasis and other intracellular infections.

## Supporting information

S1 Fig (A,B) Effect of SphK inhibitors; SKI-5C and DMS in *Leishmania promastigotes.* To evaluate the IC_50_ for SKI-5C and DMS, approximately 5 × 10^4^ *Ld* Bob cells were seeded in each well of 96-well flat bottom plates and supplemented with M199 media containing SKI-5C and DMS (200µl/well) respectively. The cells were further incubated for 2 days at 37°C and 5% CO_2_. The IC_50_ were found to be 100μM for SKI-5C and 500μM for DMS respectively in *Leishmania donovani* promastigotes. Each experiment was done in triplicates and repeated thrice.
(TIFF)

S2 Fig Effect of SphK2 inhibitor; ABC294640 in *Leishmania spp* and host macrophages. To evaluate the IC_50_ for ABC294640, approximately 6 × 10^3^ THP-1 and 5 × 10^4^ *Ld* Bob cells were seeded in each well of 96-well flat bottom plates and supplemented with RPMI and M199 media containing ABC294640 (200µl/well) respectively. For intracellular amastigotes, 1 × 10^6^ THP-1 cells, treated with 50 ng/ml of phorbol 12-myristate 13-acetate (PMA) were seeded on glass coverslip in a 6-well plate for 48h. They were infected with late log-phase *L. donovani* promastigotes and simultaneously treated with ABC294640. The cells were further incubated for 2 days at 37°C and 5% CO_2_. To determine, the intracellular parasite burdens (mean number of amastigotes per macrophage) were microscopically assessed using Giemsa staining. For axenic amastigotes, the axenically cultured forms grew optimally at a temperature of 32–33°C in a growth media with pH of 5.4. The IC_50_ were found to be 69.7μM for intracellular amastigotes and 60 μM for THP-1 macrophages. Each experiment was done in triplicates and repeated thrice.
(TIFF)

S3 Fig (A) Nucleotide sequence encoding full length sequence of *L. donovani* Sphk1 (1–2808 bp). This sequence was selected for recombinant protein generation as a fusion protein with an N-terminal 6x- Histidine-tag using the vector pET-28 a(+) vector. **(B) Cloning of *L. donovani* SphK1 in pET28a(+) vector.** PCR amplification of DNA fragment from genomic DNA of *L. donovani* using *Ld*Sphk1_BamHI and *Ld*Sphk1_XhoI with Phusion High-Fidelity DNA Polymerase. Purification of the amplified DNA fragment was done using QIAquick Gel Extraction Kit followed by digestion of purified *Ld*Sphk1 insert and the expression vector pET-28 a(+) with BamHI/XhoI restriction enzymes. The positive clones were screened by colony PCR followed by confirmation of the cloned plasmid with BamHI/XhoI restriction digestion. **(C) *rLd*Sphk1 recombinant protein purification**. Transformation of positive clone in Rossetta *E. coli* expression strain for *Ld*Sphk1 recombinant protein expression. For recombinant protein induction, the cells were induced with 1mM IPTG for 4h at 37°C in Terrific broth. Purification of recombinant protein was achieved by affinity chromatography. The *rLd*SphK1 protein was eluted after addition of 150mM imidazole solution. The eluted fractions were analysed by running on 12% SDS PAGE. **(D) The enzymatic activity (K**_**m**_ **and V**_**max**_**) of *rLd*SphK1.** Varying different concentration of NBD Sphingosine (2–10μM) and keeping *rLd*SphK1 constant (200ng), NBD-SIP levels were measured to calculate K_m_ and V_max_ of *rLd*SphK1. For this, the recombinant protein was incubated with 200μM ATP, NBD–Sphingosine (NBD-Sph) was used as a substrate and conversion of NBD-Sph to NBD-S1P was evaluated. The plate was immediately placed on the varioskan LUX Multimode Microplate Reader (Thermo fisher, Massachusetts, USA) every 5 seconds for 20 min. Readings were taken real-time after every 5 sec for 15–20 min at an excitation/emission wavelength of 490/530 nm. Standard curve of SIP was plotted. The enzymatic activities were calculated by plotting fluorescence/time vs substrate concentration followed by plotting of fluorescence/pmoles (velocity) vs substrate concentration. Finally 1/Vmax vs 1/[S] was plotted to calculate Km and Vmax according to Lineweaver-Burk plots at 37°C.
(TIFF)

S4 Fig 3D Protein Structure model comparison of *Ld*SphK1. **(A) Table showing various performance metrics for our homology model and AlphaFold (B) (i) Ramachandran plot analysis of the *Ld*SphK1 homology model.** The plot shows the stereochemical quality of the protein’s backbone dihedral angles. The analysis revealed that 81.99% of residues are in the most favoured regions, while 6.11% are classified as outliers. This, combined with a low overall MolProbity score of 1.74, confirms the high quality of the generated model. **(ii) Ramachandran plot for AlphaFold’s model** showing 82.85% residues in favoured regions. This along with the MolProbity score confirms the rationale for using the homology model.
(TIFF)

S5 Fig (A) Amino acid sequence alignment of *Ld*_SphK1 and Human_SphK1 utilizing MEGAX program (version 10.2.2). The results demonstrated considerable alignment with similar and a limited number of identical amino acids. **(B) Phylogenetic analysis of Sphingosine Kinase 1.** The evolutionary history was inferred using the Maximum Likelihood (ML) method based on the JTT + G + I + F model in MEGA XII. The tree was constructed using 1000 bootstrap replicates, and is drawn to scale, with branch lengths measured in the number of substitutions per site. The analysis clearly separates the kinetoplastid enzymes from the mammalian (host) orthologs, with Trypanosoma species used as the outgroup. **(C) 3D structural alignment of *Ld*_SphK1 and Human_SphK1 protein structures with Pymol software.** Substantial alignment between the two proteins, with an RMSD of 0.585 was observed. Furthermore, the alpha helix and beta sheets of the Human DAGKc domain (Blue) accurately aligned with the LD_DAGKc domain (Yellow).
(TIFF)

S6 Fig (A) Purification of the recombinant *human*SphK1 (*rhuman*SphK1) protein. Overexpression of 6 × His-SphK-1 (*rhSphK-1*) was induced with 1mM IPTG at an optical density (OD600) of 0.6, for 4 h at 37°C. The protein was purified using Ni-NTA agarose resin. The *rhSphK1* protein was eluted with a continuous imidazole gradient of 50mM, 100mM, 250mM, and 500mM. The protein purification was validated by 12% SDS-PAGE, followed by immunoblotting with anti-His tag antibody. A single band corresponding to ~50 kDa was observed on SDS PAGE corresponding to Human specific SphK1. **(B) The enzymatic activity (K**_**m**_ **and V**_**max**_**) of *rh*SphK1.** Varying different concentration of NBD Sphingosine (2–10μM) and keeping *rhSphK1* constant (200ng), NBD-SIP levels were measured to calculate K_m_ and V_max_ of *rhSphK1*. For this, the recombinant protein was incubated with 200μM ATP, NBD–Sphingosine (NBD-Sph) was used as a substrate and conversion of NBD-Sph to NBD-S1P was evaluated. The plate was immediately placed on the varioskan LUX Multimode Microplate Reader (Thermo fisher, Massachusetts, USA) every 5 seconds for 20 min. Readings were taken real-time after every 5 sec for 15–20 min at an excitation/emission wavelength of 490/530 nm. Standard curve of SIP was plotted. The enzymatic activities were calculated by plotting fluorescence/time vs substrate concentration followed by plotting of fluorescence/pmoles (velocity) vs substrate concentration. Finally 1/Vmax vs 1/[S] was plotted to calculate Km and Vmax according to Lineweaver-Burk plots at 37°C.
(TIFF)

S7 Fig Estimation of glutathione levels upon PF-543 treatment in *Leishmania* infected macrophages. THP-1 macrophages were cultured in six-well plates in the presence of *L. donovani* infection (MOI, 20:1) for 6h. Infected THP-1 were washed to remove non-internalized parasites and treated with PF-543 (20μM) or DL-threo-PPMP; glucosylceramide synthase inhibitor (20 μM) or SIP (10μM) for next 48h. GSH/GSSG levels were analysed using cell lysates quantified for GSG/GSSH assay. Data from two of the experiments is shown.
(TIFF)

S1 Table Primer sequences of target genes mentioned along with their annealing temperatures. (TIFF)
