# Supplementary figures and images for: Targeting Leishmania donovani Sphingosine Kinase 1 using PF-543 enhances immune response and limits parasite load
Source: PLoS Negl Trop Dis. 2026 Mar 20;20(3):e0013102. doi: 10.1371/journal.pntd.0013102 (PMC13086438; doi:10.1371/journal.pntd.0013102)

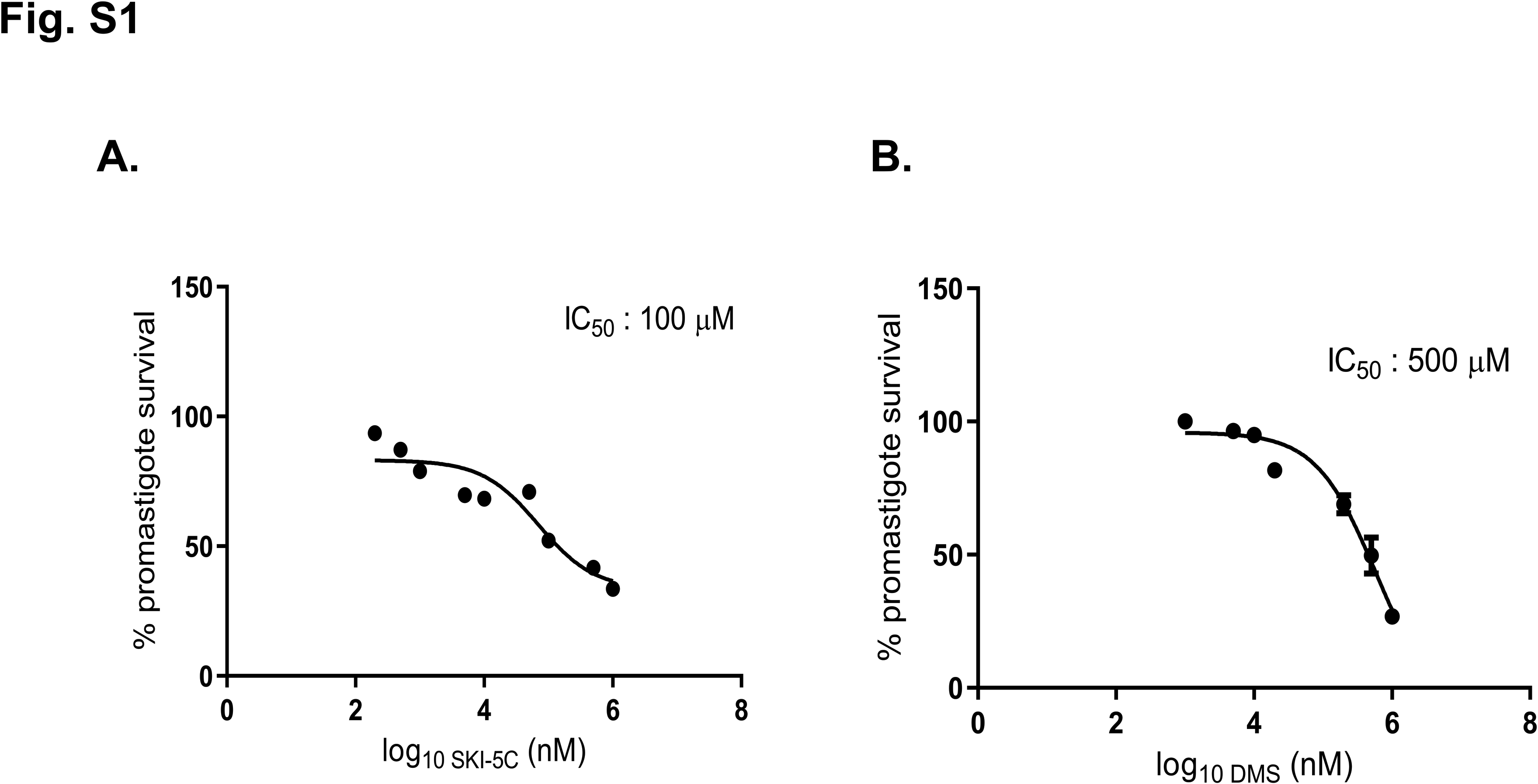

Supplement: S1 Fig — To evaluate the IC50 for SKI-5C and DMS, approximately 5 × 10^4^ Ld Bob cells were seeded in each well of 96-well flat bottom plates and supplemented with M199 media containing SKI-5C and DMS (200µl/well) respectively. The cells were further incubated for 2 days at 37°C and 5% CO2. The IC50 were found to be 100μM for SKI-5C and 500μM for DMS respectively in Leishmania donovani promastigotes. Each experiment was done in triplicates and repeated thrice. (TIFF) [file pntd.0013102.s001.tiff]

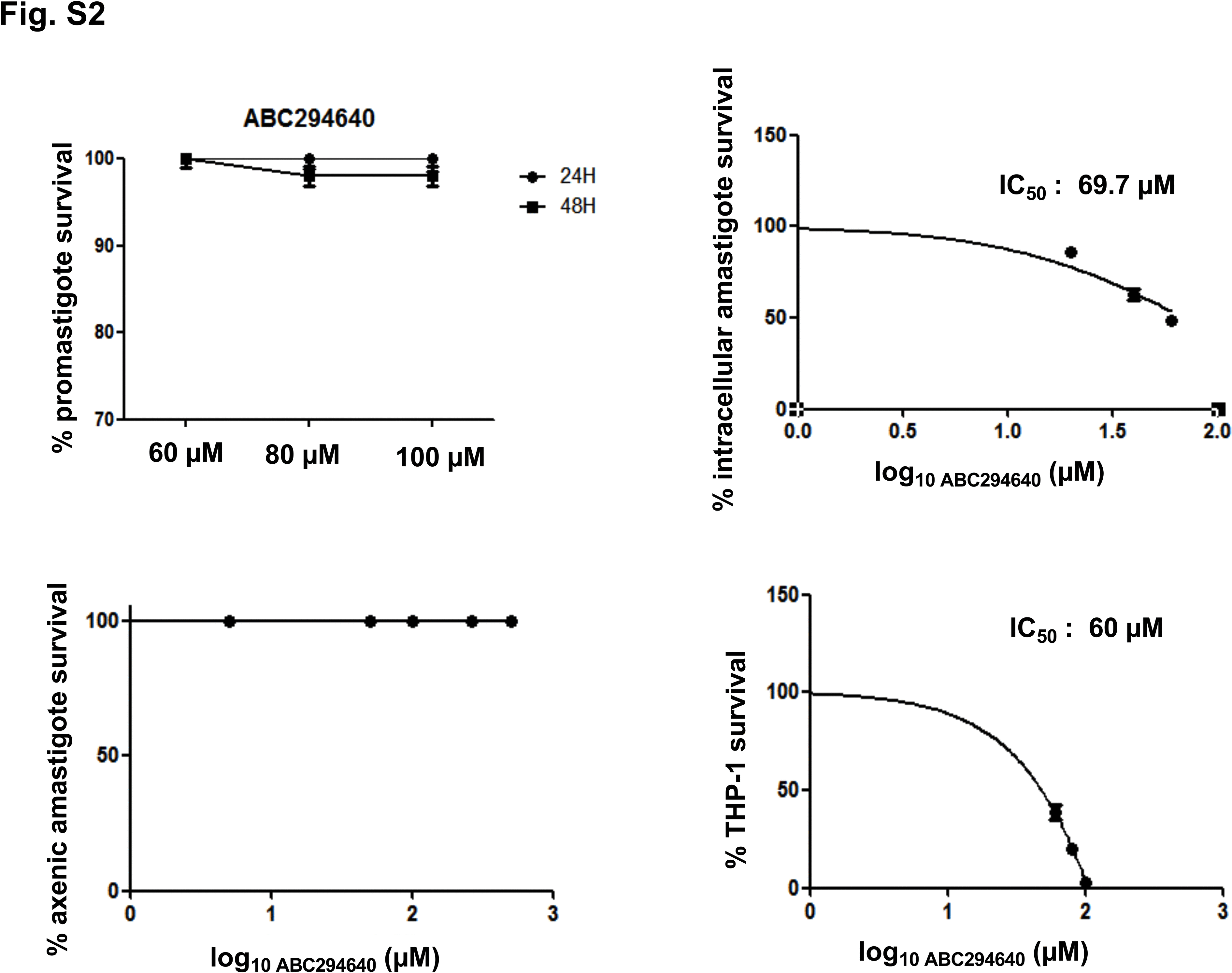

Supplement: S2 Fig — To evaluate the IC50 for ABC294640, approximately 6 × 10^3^ THP-1 and 5 × 10^4^ Ld Bob cells were seeded in each well of 96-well flat bottom plates and supplemented with RPMI and M199 media containing ABC294640 (200µl/well) respectively. For intracellular amastigotes, 1 × 10^6^ THP-1 cells, treated with 50 ng/ml of phorbol 12-myristate 13-acetate (PMA) were seeded on glass coverslip in a 6-well plate for 48h. They were infected with late log-phase L. donovani promastigotes and simultaneously treated with ABC294640. The cells were further incubated for 2 days at 37°C and 5% CO2. To determine, the intracellular parasite burdens (mean number of amastigotes per macrophage) were microscopically assessed using Giemsa staining. For axenic amastigotes, the axenically cultured forms grew optimally at a temperature of 32–33°C in a growth media with pH of 5.4. The IC50 were found to be 69.7μM for intracellular amastigotes and 60 μM for THP-1 macrophages. Each experiment was done in triplicates and repeated thrice. (TIFF) [file pntd.0013102.s002.tiff]

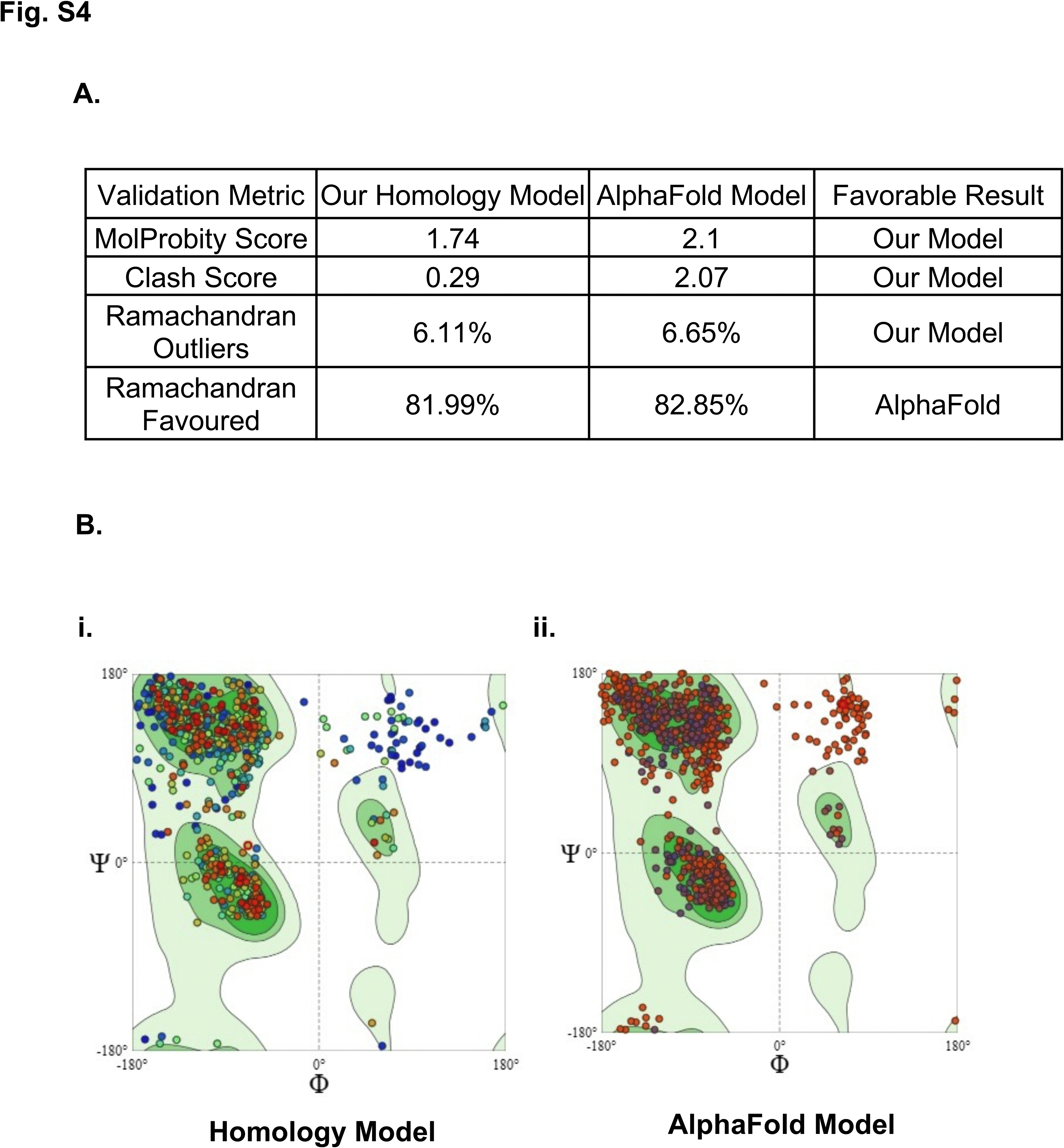

Supplement: S4 Fig — (A) Table showing various performance metrics for our homology model and AlphaFold (B) (i) Ramachandran plot analysis of the LdSphK1 homology model. The plot shows the stereochemical quality of the protein’s backbone dihedral angles. The analysis revealed that 81.99% of residues are in the most favoured regions, while 6.11% are classified as outliers. This, combined with a low overall MolProbity score of 1.74, confirms the high quality of the generated model. (ii) Ramachandran plot for AlphaFold’s model showing 82.85% residues in favoured regions. This along with the MolProbity score confirms the rationale for using the homology model. (TIFF) [file pntd.0013102.s004.tiff]

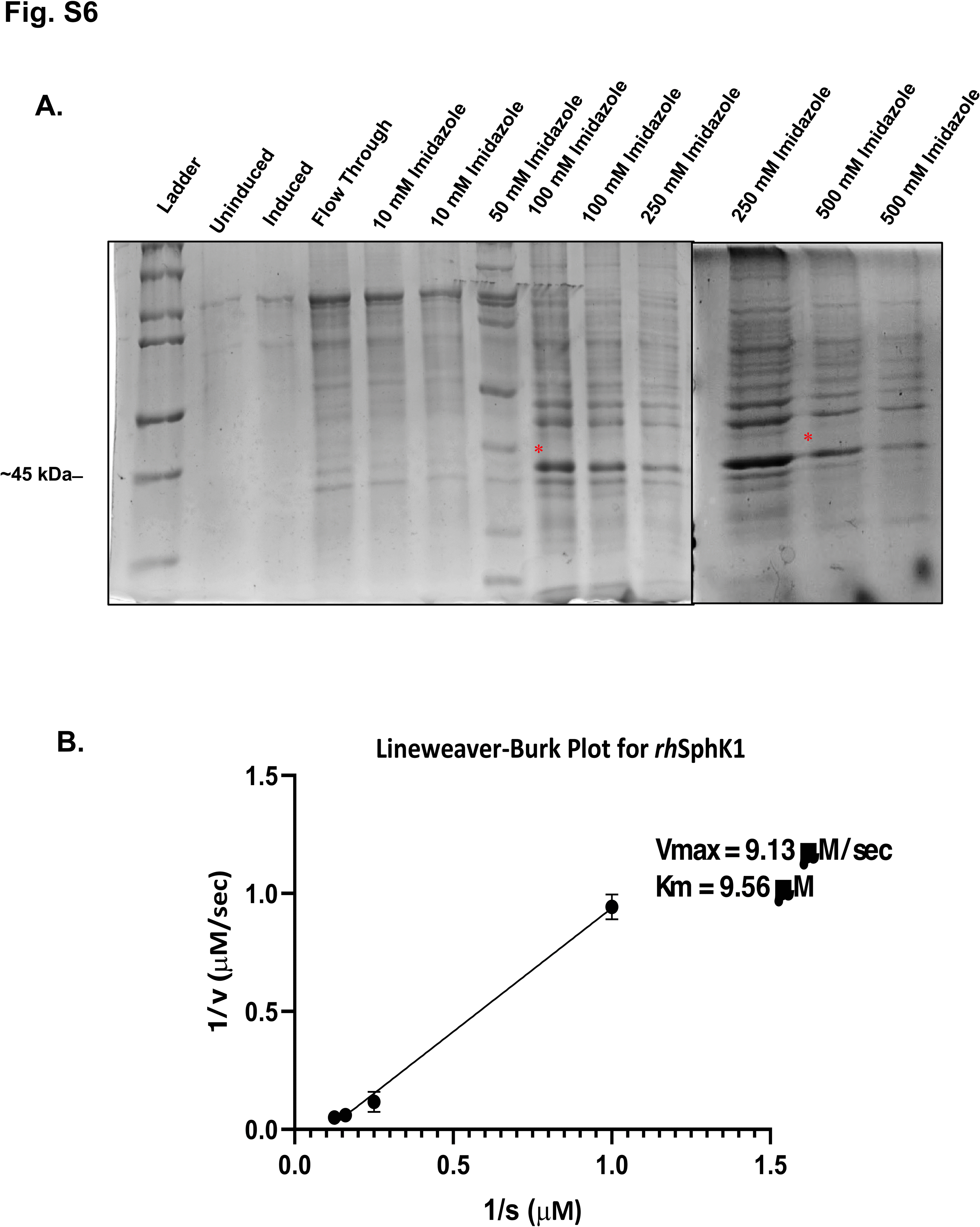

Supplement: S6 Fig — Overexpression of 6 × His-SphK-1 (rhSphK-1) was induced with 1mM IPTG at an optical density (OD600) of 0.6, for 4 h at 37°C. The protein was purified using Ni-NTA agarose resin. The rhSphK1 protein was eluted with a continuous imidazole gradient of 50mM, 100mM, 250mM, and 500mM. The protein purification was validated by 12% SDS-PAGE, followed by immunoblotting with anti-His tag antibody. A single band corresponding to ~50 kDa was observed on SDS PAGE corresponding to Human specific SphK1. (B) The enzymatic activity (Km and Vmax) of rhSphK1. Varying different concentration of NBD Sphingosine (2–10μM) and keeping rhSphK1 constant (200ng), NBD-SIP levels were measured to calculate Km and Vmax of rhSphK1. For this, the recombinant protein was incubated with 200μM ATP, NBD–Sphingosine (NBD-Sph) was used as a substrate and conversion of NBD-Sph to NBD-S1P was evaluated. The plate was immediately placed on the varioskan LUX Multimode Microplate Reader (Thermo fisher, Massachusetts, USA) every 5 seconds for 20 min. Readings were taken real-time after every 5 sec for 15–20 min at an excitation/emission wavelength of 490/530 nm. Standard curve of SIP was plotted. The enzymatic activities were calculated by plotting fluorescence/time vs substrate concentration followed by plotting of fluorescence/pmoles (velocity) vs substrate concentration. Finally 1/Vmax vs 1/[S] was plotted to calculate Km and Vmax according to Lineweaver-Burk plots at 37°C. (TIFF) [file pntd.0013102.s006.tiff]

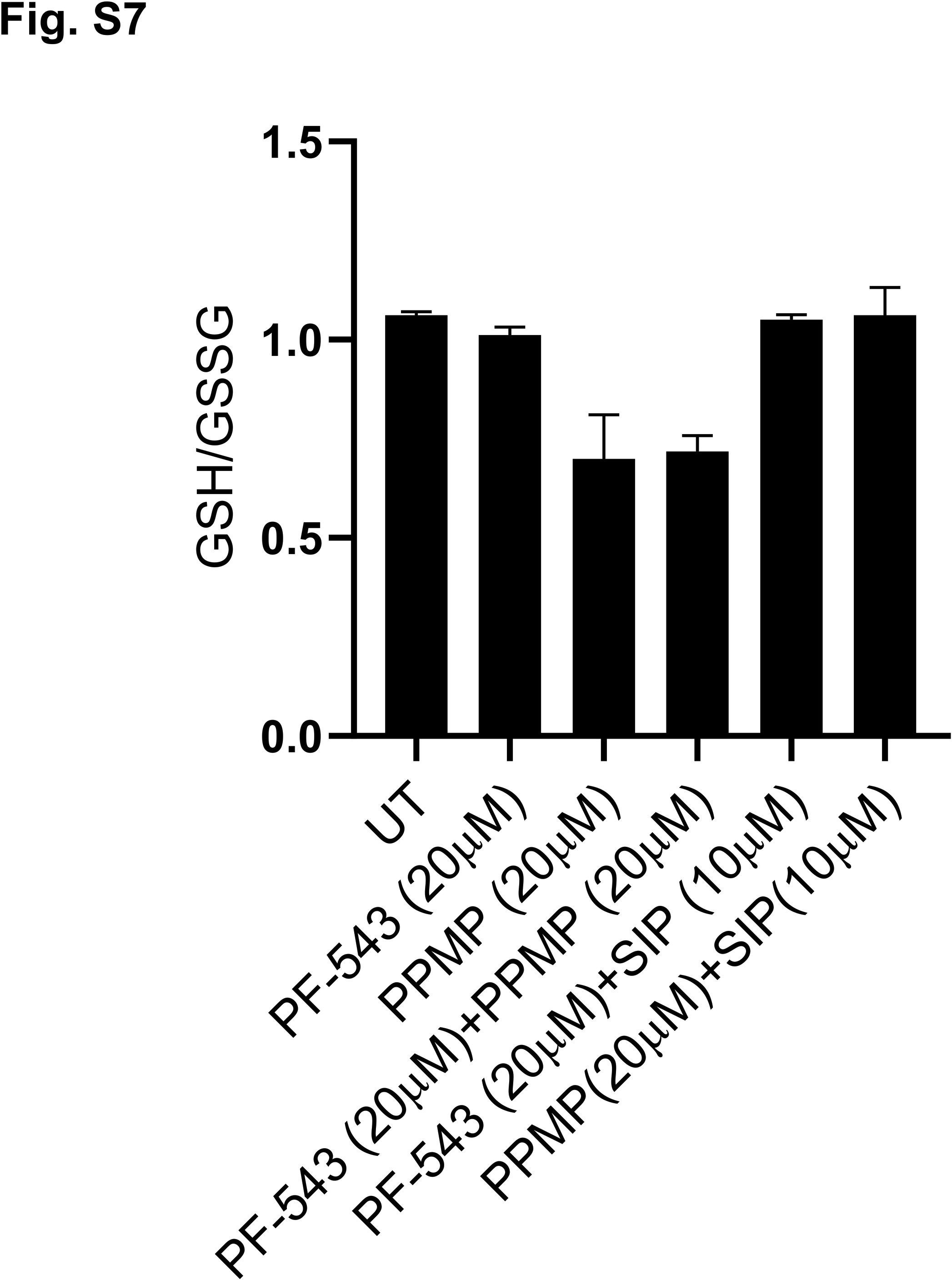

Supplement: S7 Fig — THP-1 macrophages were cultured in six-well plates in the presence of L. donovani infection (MOI, 20:1) for 6h. Infected THP-1 were washed to remove non-internalized parasites and treated with PF-543 (20μM) or DL-threo-PPMP; glucosylceramide synthase inhibitor (20 μM) or SIP (10μM) for next 48h. GSH/GSSG levels were analysed using cell lysates quantified for GSG/GSSH assay. Data from two of the experiments is shown. (TIFF) [file pntd.0013102.s007.tiff]

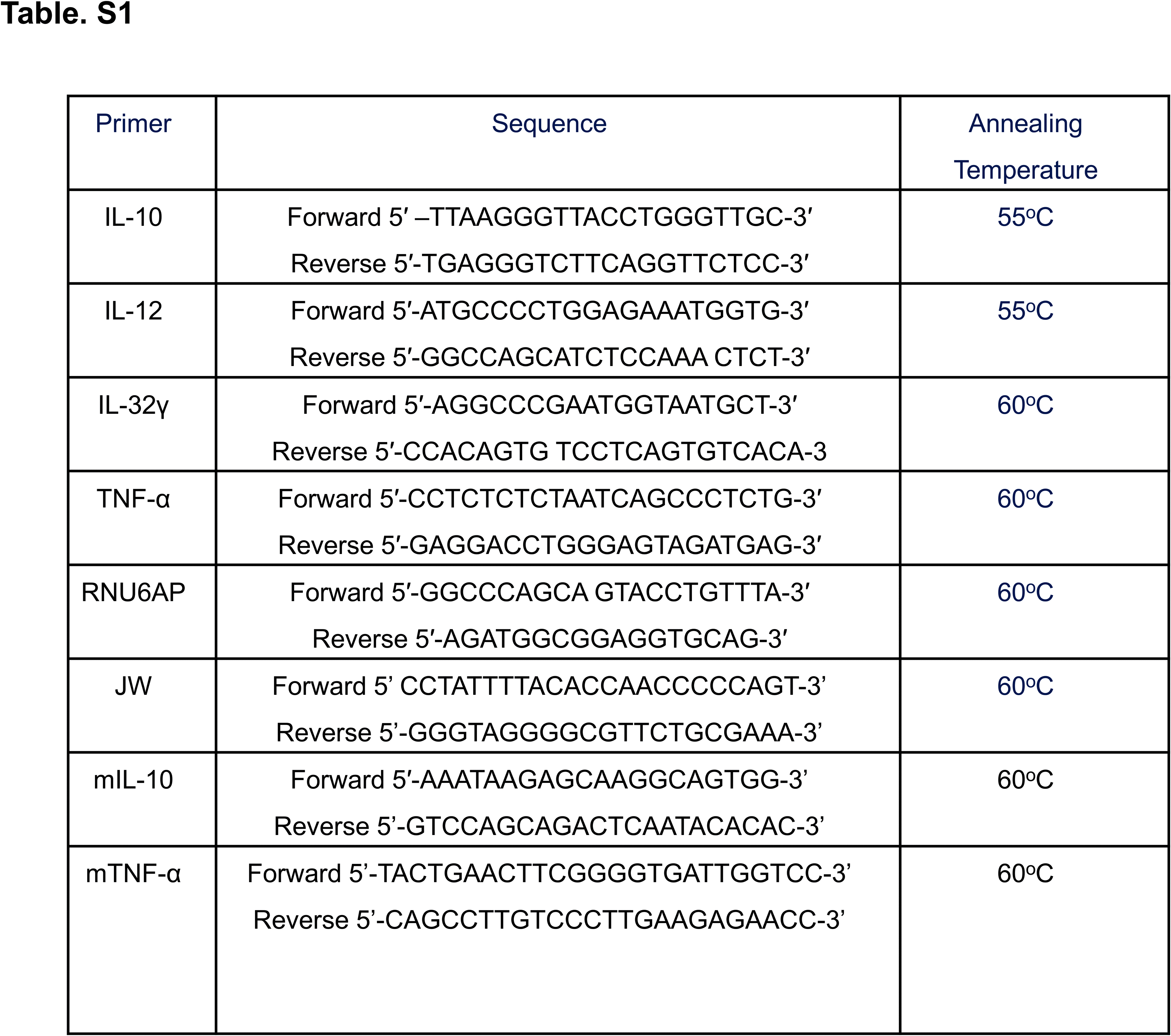

Supplement: S1 Table — (TIFF) [file pntd.0013102.s008.tiff]
